# The cryo-EM structure and physical basis for anesthetic inhibition of the THIK1 K2P channel

**DOI:** 10.1073/pnas.2421654122

**Published:** 2025-04-03

**Authors:** Elena B. Riel, Weiming Bu, Thomas T. Joseph, Leila Khajoueinejad, Roderic G. Eckenhoff, Paul M. Riegelhaupt

**Affiliations:** ^a^Department of Anesthesiology, Weill Cornell Medical College, New York, NY 10065; ^b^Department of Anesthesiology and Critical Care, University of Pennsylvania, Philadelphia, PA 19104

**Keywords:** cryoEM, K2P, anesthetics, ion channel, THIK1

## Abstract

THIK1 tandem pore domain (K2P) potassium channels regulate microglial surveillance of the central nervous system and responsiveness to inflammatory insults. With microglia recognized as critical to the pathogenesis of neurodegenerative diseases, THIK1 channels are putative therapeutic targets to control microglia dysfunction. While THIK channels can principally be distinguished from other K2Ps by their distinctive inhibitory response to volatile anesthetics (VAs), molecular details governing THIK channel gating remain largely unexplored. Here, we report a 3.2 Å cryo-electron microscopy structure of the THIK1 channel in a closed conformation. A central pore gate located directly below the THIK1 selectivity filter is formed by inward-facing TM4 helix tyrosine residues that occlude the ion conduction pathway. VA inhibition of THIK requires closure of this central pore gate. Using a combination of anesthetic photolabeling, electrophysiology, and molecular dynamics simulation, we identify a functionally critical THIK1 VA binding site positioned between the central gate and a structured section of the THIK1 TM2/TM3 loop. Our results demonstrate the molecular architecture of the THIK1 channel and elucidate critical structural features involved in regulation of THIK1 channel gating and anesthetic inhibition.

## Significance

THIK1 potassium channel activity controls the function of microglia, immune cells of the central nervous system. As microglia are involved in the pathogenesis of neurodegenerative diseases, THIK1 is an intriguing therapeutic target to modulate microglial function. Molecular details governing THIK1 channel gating are largely unknown and we utilized cryo-electron microscopy to solve the structure of the THIK1 channel. We identify unique structural features that differentiate THIK1 from related potassium channels and locate a functionally important site where an inhibitory volatile anesthetic drug binds to the THIK1 channel. These insights lay the groundwork for future studies aimed at development of THIK1-specific pharmacology, to target microglia in vivo.

Tandem pore domain (K2P) potassium channels play vital roles across human physiology, conducting noninactivating potassium currents that set cellular resting membrane potentials. The TWIK-related Halothane Inhibited K^+^ channel (THIK1), named for the early observation that halothane and other volatile anesthetic (VA) agents inhibit THIK1 currents (1), is a K2P channel expressed in microglia (2), primary immune cells of the central nervous system (CNS). Tonic THIK1 activity appears to modulate normal microglial immune surveillance behavior in the CNS (3). In response to inflammatory or infectious neuronal cues, THIK1 mediated efflux of potassium ions from the microglial cytoplasm modulates activation of the NLRP3 inflammasome (4) and pharmacologic or genetic disruption of THIK1 activity decreases inflammasome-mediated production of the proinflammatory cytokine IL-1β (2, 5). Manipulation of THIK1 channel activity to control the NLRP3 inflammasome has been proposed as an avenue for treatment of Alzheimer’s disease and other neurodegenerative disorders (6), where CNS inflammation is a causative cue (7). General anesthetic drugs are known to impact immune cell function (8, 9) and microglial mediated CNS inflammation has been shown to contribute to both cognitive decline after surgery and postoperative delirium (10, 11). Whether transient inhibition of microglial THIK1 activity during routine intraoperative VA exposure has a meaningful impact on CNS immune sensitivity or neuroinflammation is an open and intriguing question.

Tools to address the role of the THIK1 channel in specific microglial functions remain limited by a lack of knowledge of THIK1 channel structure, gating, and pharmacology. While no THIK1-specific chemical activators have been described, THIK1 is potentiated by certain lipids, including arachidonic acid (1, 12) and long chain CoA (13). Recently described high affinity THIK1 inhibitors promise to be valuable tools to modulate THIK1 activity in vivo (2, 14), though the mechanism by which these drugs modulate THIK channel function remains unexplored. Similarly, the molecular basis for VA inhibition of THIK1 is also unknown. While all other VA modulated K2P channels are potentiated by anesthetics (15–17), THIK1 is uniquely inhibited by this class of drugs, suggesting a distinct mechanism responsible for the effect of VAs of THIK1 channel function.

To define the molecular basis for THIK1 gating and inhibition by VAs, we solved a cryo-electron microscopy (cryo-EM) structure of the THIK1 channel, identifying unique structural features that underlie THIK1 gating. Our 3.2 Å THIK1 structure is in a putative closed conformation, due to the presence of an occlusive pore gate constituted by a pair of inward-facing TM4 tyrosine residues positioned directly below the potassium selectivity filter. We find that VA inhibition of THIK1 requires closure of this occlusive gate. Utilizing a combination of VA photolabeling, electrophysiology, and molecular dynamics simulation, we identify and characterize THIK1 anesthetic binding sites, including one that lies at the center of a functionally critical hydrophobic pocket formed by residues from the TM2, TM3, and TM4 helices and a uniquely structured section of the distal THIK1 TM2/TM3 loop. Mutations to residues within the anesthetic binding site or the structured portion of the TM2/TM3 loop all lead to potentiated THIK1 currents and diminish or eliminate isoflurane inhibition of THIK1. Our findings define the structural architecture of the THIK1 channel, identify functionally critical regions of the THIK1 structure involved in gating, and elucidate a potential mechanism for VA inhibition of THIK channel function.

## Results

**Fig. 1.**
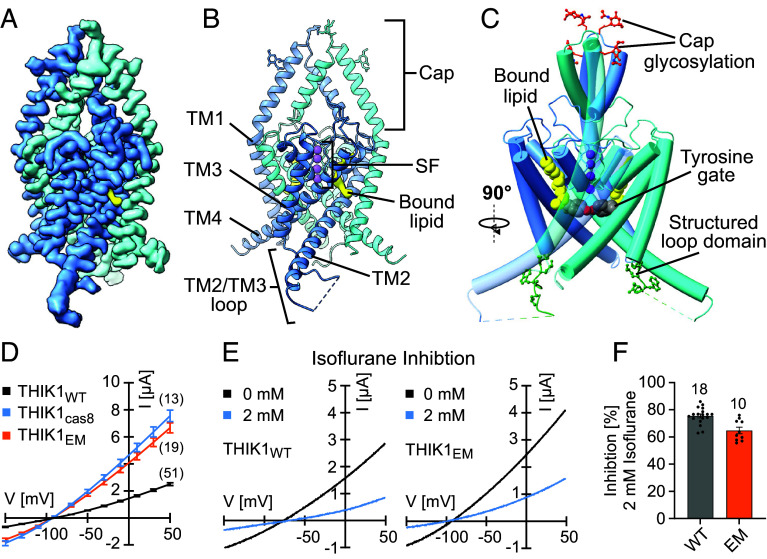
THIK1 cryo-EM structure. (*A*) Cryo-EM map and (*B*) molecular model of the THIK1 channel structure (monomer A blue, monomer B teal). The position of a buried lipid-like density within the channel structure is highlighted (yellow). (*C*) Overview of the unique features in the THIK1 structure, including glycosylation of the N59 and N65 residues on the THIK1 cap (orange red), the bound lipid buried behind the THIK1 selectivity filter (yellow), a closed tyrosine gate within the pore vestibule (gray) and a structured M2/M3 loop domain (green). Potassium ions are depicted as purple spheres. (*D*) IV curves generated by TEVC recordings (*X. laevis*) from full-length THIK1 (THIK1_WT_, black) or THIK1 C-terminal truncation constructs at either the endogenous caspase 8 cleavage site (THIK1_cas8_, blue) or the introduced C-terminal truncation in the THIK1 Cryo-EM construct (THIK1_EM_, orange). (*E*) Exemplary current traces (TEVC, *X. laevis*) of THIK1_WT_ and THIK1_EM_ basal currents (black) and response to treatment with 2 mM isoflurane (blue) and (*F*) Isoflurane inhibition (%) of WT and THIK1_EM_ construct calculated from recordings as shown in (*E*). Values and statistics are listed in *SI Appendix,* Table S2 and error bars represent mean ± SEM.

To facilitate cryo-EM structure determination and anesthetic photolabeling studies, we purified a C-terminally truncated version of THIK1 (KCNK13), bearing residues 1 to 309 of the THIK1 protein sequence (*SI Appendix,* Fig. S1). In two electrode voltage clamp (TEVC) electrophysiology studies (Fig. 1*D*), the THIK1_EM_ C-terminally truncated construct exhibited enhanced current density relative to the full-length WT THIK1 channel, comparable to a physiological THIK1 truncation at the caspase 8 recognition site within the THIK1 C-terminus (18). The C-terminally truncated THIK1_EM_ construct retained VA sensitivity (Fig. 1 *E* and *F*) and could be readily purified after expression in HEK293 cells (*SI Appendix,* Fig. S1 *A* and *B*). Using cryo-EM, we solved a 3.2 Å structure of this THIK1_EM_ channel construct (Fig. 1*A* and *SI Appendix,* Figs. S1 and S2).

The overall structure of the THIK1 channel follows the canonical K2P architecture, featuring a dimeric channel assembly with a domain-swapped cap domain at the extracellular face of the channel, positioned above a central pore formed by the TM2 and TM4 helices (Fig. 1 *A* and *B*). The cap domain of THIK1 contains two predicted N-glycosylation sites at Asp59 and Asp65 and in our THIK1 cryo-EM map we observe density consistent with glycosylation of both sites (Fig. 1 *A*–*C* and *SI Appendix,* Fig. S3*E*). Similar to reports for other K2Ps (19–21), we find that glycosylation of THIK1 is crucial for promoting plasma membrane trafficking or preventing cellular degradation in mammalian cells, as GFP tagged THIK1 WT, N59Q, or N65Q single mutant channels were readily detectable at the HEK293 cell surface while a GFP tagged THIK1 N59Q N65Q double mutant channel completely lacked GFP fluorescent signal (*SI Appendix,* Fig. S3 *F* and *G*). Disruption of one or both glycosylation sites had no impact on THIK1 current density when measured by two electrode voltage clamp (TEVC) in *Xenopus* oocytes (*SI Appendix,* Fig. S3*H*), indicating that 1) glycosylation does not appreciably alter THIK1 channel function and 2) the influence of glycosylation on THIK1 trafficking or degradation observed in mammalian cells is not equivalently preserved in the *Xenopus* oocytes system, a pattern that mirrors prior results for Shaker potassium channels (22).

Several other features of the THIK1 channel structure are notable and distinguish THIK1 from other K2Ps (Fig. 1*C*). These include an inner pore gate positioned directly below the THIK1 selectivity filter, a uniquely structured section of the loop between the TM2 and TM3 helices, and a lipid density buried behind the selectivity filter within the core of the THIK1 protein (Fig. 1*C* and *SI Appendix,* Fig. S3*C*).

### An Occlusive Gate at the Center of the THIK1 Pore.

**Fig. 2.**
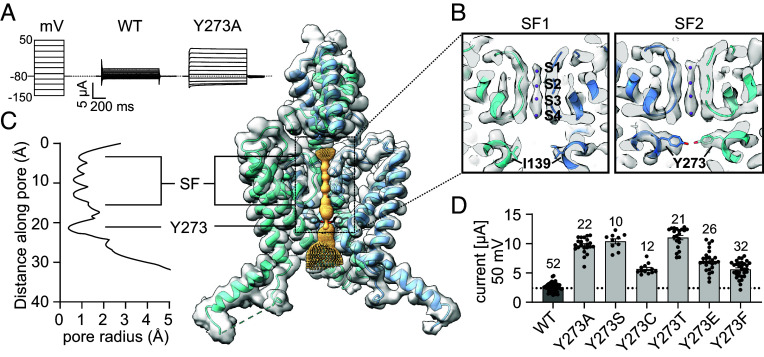
The THIK1 tyrosine pore gate. (*A*) TEVC current traces (*X. laevis*) from step protocol recordings in 20 mV steps from −150 mV to 50 mV (*Left*) for THIK1 WT (*Middle*) and Y273A mutant channels (*Right*). (*B*) Overlay of the postprocessed cryo-EM map and THIK1 model, showing the SF and nearby gating residues Y273 (TM4) and I139 (TM2). At this visualization threshold, four potassium ions are observed within the filter (S1 to S4, purple). (*C*) THIK1 pore profile as determined by HOLE software, with narrowing at the Y273 gate and selectivity filter regions noted. (*D*) TEVC currents (*X. laevis*) at 50 mV holding potential, for THIK1 WT (black) and Y273 mutants (light gray). Values and statistics are listed in *SI Appendix,* Tables S1 and S2 and error bars represent mean ± SEM.

At its intracellular face, the THIK1 channel pore lies open to the cytoplasm, lacking an occlusive cytoplasmic pore gate like those found in structures of TASK1 or TASK2 K2P channels (23, 24) (*SI Appendix,* Fig. S4*B*). While the unobstructed cytoplasmic pore vestibule in THIK1 is somewhat reminiscent of the open pore structures observed in the mechanosensitive K2Ps TREK1, TREK2, and TRAAK (25–28) (*SI Appendix,* Fig. S4*D*), THIK1 features a unique central gating element absent in all other K2P channels. Inwardly opposed Tyr273 sidechains arise from the pore-lining TM4 helixes (Figs. 1*C* and 2 *B* and *C*) and meet within the pore to form a central occlusive constriction ~6 Å below the THIK1 selectivity filter (Fig. 2*B* and *SI Appendix,* Fig. S4 *A* and *B*). This occlusive tyrosine gate (Y-gate) is positioned much closer to the potassium selectivity filter than either the TASK1 intracellular X-gate occlusion (24) or the TASK2 K245 internal proton sensor gate (23) (*SI Appendix,* Fig. S4*B*). In the presumed closed conformation we observe in the THIK1 structure, the Y-gate tyrosine residues limit the size of the ion conduction pathway to a diameter of less than 2 Å (Fig. 2*C*), precluding passage of potassium ions to the selectivity filter and dividing the inner pore into a large lower vestibule and a much smaller hydrophilic chamber directly below the selectivity filter (Fig. 2 *B* and *C* and *SI Appendix,* Fig. S4*A*).

To explore the functional role of the Y-gate in controlling THIK1 activity, we created a series of point mutants at Tyr273 and examined the electrophysiological properties of the resultant channels. In every case, mutagenesis of Tyr273 led to an increase in current density, confirming that the Y-gate is a critical regulatory element limiting potassium flow through the THIK1 channel. Amino acid size seems to play a significant role in determining the functional effect of Y273 mutants, as insertions of small amino acid residues at Tyr273 (Y273A, Y273S, Y273T) led to the largest changes in THIK1 current density (Fig. 2 *A* and *D* and *SI Appendix,* Fig. S4*C*). This result is consistent with the structural observation that the Y-gate produces a steric hindrance to ionic movement through the pore, with mutations that widen the size of the Y-gate occlusion leading to an expected increase in channel activity. However, the chemical nature of the inserted amino acid was also determinative. The gain of function observed in the THIK1 Y273F channel best exemplifies this notion (Fig. 2*D*), as steric considerations only minimally differ between THIK1 WT and the Y273F mutant, while the change in residue polarity is the more critical difference in the Y273F mutant. The difference in the functional impact of the Y273S versus Y273C mutants (Fig. 2*D* and *SI Appendix,* Fig. S4*C*) is another similar example. The activity of the Y273C mutant was not modified by treatment with the chemical reducing agent DTT (*SI Appendix,* Fig. S4*E*), suggesting that the inserted cysteines at the Y-gate do not form a spontaneous disulfide bond. The divergent effects of the equivalently sized Y273C and Y273S residues thus likely reflect the relatively less polar and electronegative sulfhydryl of the Y273C, as compared to the hydrophilic hydroxyl group in the Y273S mutant.

### Disruption of THIK1 Y-Gate Function Reduces the Inhibitory Effect of Anesthetics.

**Fig. 3.**
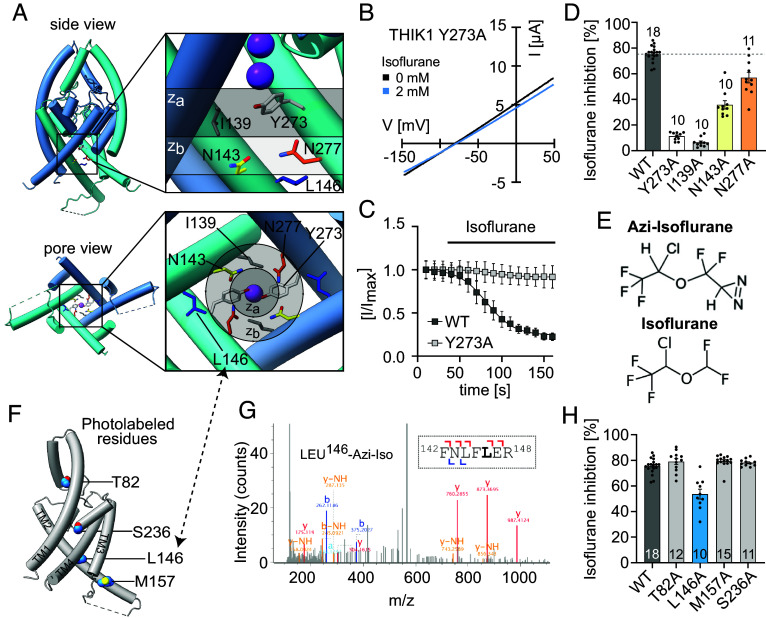
Identification of a functionally critical THIK1 isoflurane inhibitory sites. (*A*) Side and pore views of THIK1, with the Y273 (white), I139 (gray), N143 (yellow), and N277 (orange) residues that contribute to the central Y-gate displayed as sticks. *Insets* show the residue arrangement of Y273 and I139 in an upper plane z_a_ and the asparagine ring formed by the residues N143 and N277 symmetrically arranged below, in lower plane z_b_. The lower halves of the front-facing TM1 and TM2 helices are hidden in the side view panel, to allow for visualization of the residue at the central pore gate. (*B*) Exemplary current traces (TEVC, *X. laevis*) of the THIK1 Y273A mutant channel before (black) and after isoflurane (blue, 2 mM) application and (*C*) time course of THIK1 WT (black) or Y273A mutant (gray) channel current (at 0 mV holding) after exposure to 2 mM isoflurane. (*D*) Isoflurane inhibition (%) of basal currents for THIK1 WT or mutants. (*E*) Chemical structures of isoflurane and its photolabeling analog azi-isoflurane. (*F*) Sites of azi-isoflurane photolabeling, mapped on the THIK1 monomer structure. (*G*) Mass spectrum of the THIK1 Leu146 azi-isoflurane adducted peptide, with peptide fragmentation pattern shown in the *Inset*. (*H*) Isoflurane (2 mM) inhibition (%) of THIK1 WT (black) or THIK1 mutant channels containing a single alanine substitution at the azi-isoflurane photolabeled sites. Values and statistics are listed in *SI Appendix,* Table S2 and error bars represent mean ± SEM.

As the Y-gate appears to be a critical element responsible for THIK1 channel closure, we tested whether VA inhibition of THIK1 involves this occlusive gate. We found that mutations that reduce the size of the Y-gate Tyr273 residue led to a near complete loss of THIK1 inhibition by the VA isoflurane (Fig. 3 *B*–*D* and *SI Appendix,* Fig. S5 *A* and *B*), indicating that VA inhibition of THIK1 requires Y-gate closure. In support of this assertion is the observation that only the conservative Y273F mutant, which should still maintain the necessary sidechain size to occlude the pore, retained isoflurane sensitivity (*SI Appendix,* Fig. S5 *A* and *B*). While Tyr273 is the critical residue responsible for occluding the center to the THIK1 pore in the closed state, a neighboring TM2 Ile139 residue plays a complementary role, sealing the lateral edges of the conduction pathway (Figs. 2*B* and 3*A*, plane z_a_). The THIK1 I139A mutant exhibits a similarly large increase in basal current density and near complete loss of isoflurane sensitivity (Fig. 3*D* and *SI Appendix,* Fig. S5 *A* and *C*), recapitulating the effect of mutations to the Y-gate Tyr273. These results indicate that closure of the Y-gate, with consequent occlusion of the intracellular ion conduction pathway, are central to the mechanism by which VAs inhibit THIK1 channel function

In the closed Y-gate conformation, the diameter of the THIK1 pore is too small for hydrated potassium ions to cross the Y-gate and reach the selectivity filter (Fig. 2*C* and *SI Appendix,* Fig. S4*B*). For the channel to convert to an open and conductive conformation, the Tyr273 residues should be required to rotate or translate out of the ion conduction pathway. As the hydrophilic nature of the Tyr273 residue appears to influence THIK1 function, we searched for polar residues in the vicinity of Tyr273 that could serve as interacting partners to stabilize a putative open conformation. This directed our focus to a fourfold symmetric polar ring of asparagine residues positioned directly below the Y-gate (plane z_b_, Fig. 3*A*). This N-ring, formed by the Asn143 residues from TM2 and Asn277 residues from TM4 (Fig. 3*A*), is located ~5.5 Å below the Y-gate (intrasubunit Asn143 to Tyr273 distance 5.8 Å, Asn277 to Tyr273 distance 5.3 Å), slightly too distant from the Y273 residues to form hydrogen bonds interactions in the closed state conformation. We mutated each N-ring asparagine to alanine and found that both the N277A and N143A mutants reduced THIK1 sensitivity to isoflurane (Fig. 3*D*), suggesting that the N-ring residues modulate Y-gate opening and closure to control THIK1 channel function. Despite the symmetrical arrangement of the N-ring asparagines and their similar effects on VA sensitivity, only the TM4 N277A mutant enhanced THIK1 basal function (*SI Appendix,* Fig. S5*C*), suggesting a lack of equivalence in the role of these two asparagine residues in modulating THIK1 gating. Isoflurane sensitivity was reduced more than 50% in the N143A mutant whereas basal currents size was unaffected, indicating that perturbation of the N143 residue does not globally alter THIK1 gating but may rather play a contextually specific role in the mechanism of THIK1 VA inhibition.

### Anesthetic Photolabeling Identifies a Functionally Critical VA Binding Site.

To directly identify putative VA binding sites in the THIK1 channel, we utilized a nonbiased anesthetic photolabeling approach previously shown to successfully define VA modulatory sites in TREK1 (17) and several other ion channels (29–31). We photolabeled the detergent-purified THIK1_EM_ channel protein with azi-isoflurane, a diazirine-containing photoreactive analog of the clinically relevant VA isoflurane (Fig. 3*E*). Azi-isoflurane has previously been shown to mimic the positive modulatory effect of isoflurane at TREK1 K2P channels (17) and we find that application of azi-isoflurane similarly mimics the inhibitory effect of isoflurane on THIK1 channel currents, albeit with a lower potency (*SI Appendix,* Fig. S5*D*).

Bottom–up mass spectrometry (MS) of proteolyzed peptide fragments from the azi-isoflurane photolabeled protein positively identified 89% of the THIK1 _EM_ amino acid sequence (*SI Appendix,* Fig. S5*E*) and we found evidence of adduction of azi-isoflurane at four sites: Thr82 within the lower region of the cap domain, Leu146 and Met157 within the middle of TM2, and Ser236 at the base of the second pore helix (Fig. 3 *F* and *G* and *SI Appendix,* Fig. S9). Upon repeating azi-isoflurane photolabeling in the presence of a 100-fold excess of unmodified isoflurane (as a competitive inhibitor), only the Met157 residue remained labeled. Isoflurane protected the THIK1 Thr82, Leu146, and Ser236 sites from azi-isoflurane labeling, indicating that the clinically relevant VA exhibits specificity for these protein regions. The nonspecific labeling of M157 within the distal TM2 helix mirrors results of a prior photolabeling study, where a nonspecific azi-isoflurane binding site was similarly present in the distal TM2 of TREK1 (17).

To validate the functional relevance of the identified isoflurane binding sites, we introduced alanine mutations at each photolabeled residue and examined the isoflurane sensitivity of the resultant channels. Only mutation at the L146 site within the middle of TM2 led to a change in isoflurane sensitivity, where we observed an increase in THIK1 current density and a ~30% loss of isoflurane inhibition (Fig. 3*H*). This degree of decrease in isoflurane sensitivity matches the partial loss of isoflurane responsiveness we observe after disruption of the N-ring residues (Fig. 3*D*), both of which are positioned in close proximity to the L146 binding site (Fig. 3*A*). We therefore focused our interest on the region surrounding L146 as a functionally critical site for VA modulation of THIK1.

**Fig. 4.**
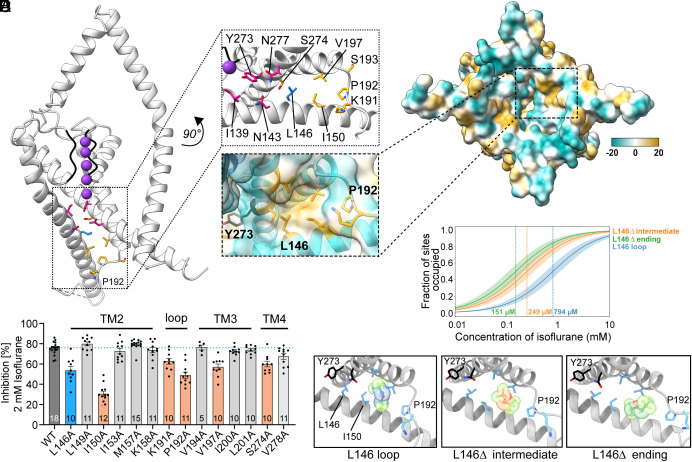
Functional validation and molecular dynamics studies of the THIK1 isoflurane binding pocket. (*A*) Side view of a THIK1 monomer structure, highlighting residues that form the central pore gate (pink), the photolabeled residue L146 (blue), and additional residues occupied by isoflurane during MD simulation (orange). The *Inset* shows an enlarged view of this pocket, rotated by ~90°. (*B*) Map of the cytoplasmic face of THIK1, showing molecular lipophilicity potential calculated in ChimeraX. The enlarged *Inset* shows the equivalent view as in panel (*A*), highlighting the hydrophobic pocket that surrounds the anesthetic binding site. (*C*) Isoflurane inhibition (%) of THIK1 currents (TEVC, *X. laevis*) for WT (black) or mutant THIK1 channels. Mutants that result in a statistically significant decrease in isoflurane sensitivity are highlighted in orange, the photolabeled residue L146 in blue. Values and statistics are listed in *SI Appendix,* Table S2 and error bars represent mean ± SEM. (*D*) Alchemical free energy perturbation titration curve, showing fraction of protein sites occupied as a function of isoflurane concentration. The unstructured TM2/3 loop was modeled using SuperLooper2 software (L146 loop) or deleted (L146 ∆). (*E*) Initial poses for the three alchemical free energy perturbation calculations shown in (*B*). L146_∆ “intermediate” and “end” poses are taken from an intermediate and end frames of the equilibration MD simulation prior to free energy perturbation calculations.

To identify additional neighboring THIK1 residues involved in VA binding and predict the pose of a bound isoflurane molecule near the L146 site, we performed all-atom MD simulations of the THIK1 channel in the presence of isoflurane. A molecule of isoflurane was placed near the L146 residue and after energy minimization and equilibrium MD simulations, we identified several additional residues that remain occupied by the isoflurane molecule (within 8 Å distance) during the simulations (Fig. 4*A* and *SI Appendix,* Table S3). Across two independent MD productions, isoflurane occupied overlapping residues that constitute a hydrophobic pocket on the cytoplasmic face of THIK1, formed by sections of the TM2, TM3, and TM4 helices as well as a short, structured span of the distal TM2/TM3 loop (Fig. 4 *A* and *B*). Corresponding alanine scanning mutagenesis experiments support a role for these residues in mediating the inhibitory effect of isoflurane, as mutations to residues facing into the hydrophobic pocket surrounding the L146 residue attenuated THIK1 isoflurane sensitivity while mutations to neighboring residues that face away from this pocket had no impact on isoflurane response (Fig. 4*C* and *SI Appendix,* Fig. S7).

We next performed alchemical free energy perturbation (AFEP) calculations to evaluate the avidity of isoflurane binding within this hydrophobic pocket. Using three distinct THIK1-bound isoflurane poses derived from the equilibrium MD simulations (Fig. 4*E*), we calculated binding Gibbs free energies of −4.4 to −5.3 kcal/mol, corresponding to K_D_ values from 151 to 794 µM (Fig. 4*D* and *SI Appendix,* Table S3). These calculated K_D_ values fall within clinically relevant concentrations of isoflurane (32) and are consistent with the experimentally determined low-affinity inhibitory effect of VAs on THIK1 function (1). As binding free energy calculations for low-affinity ligands like isoflurane are sensitive to the initial pose of the drug, the relatively large range of K_D_ values across the three calculations is likely a reflection of the differing starting poses used for the calculations.

### A Structured TM2/TM3 Loop Domain Modulates THIK1 Gating and Anesthetic Inhibition.

**Fig. 5.**
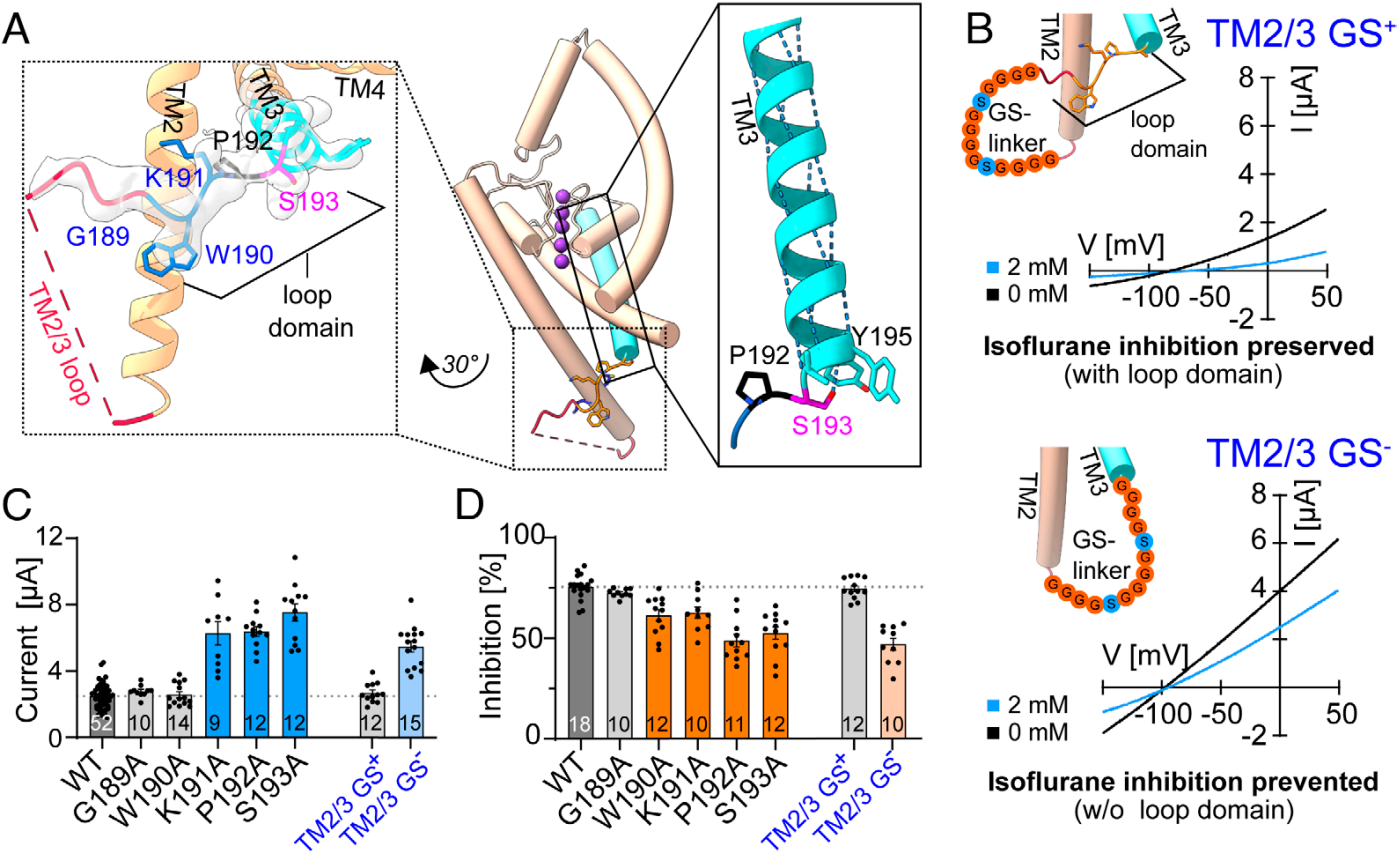
A unique TM2/TM3 loop domain regulates THIK1 function. (*A*) Side view of the THIK1 monomer, with enlarged views of the TM2/TM3 loop (*Left*) and distal TM3 helix (*Right*). An overlay of the EMReady postprocessed map is shown (*Left Inset*), overlaid with the model of the structured region of the loop. Backbone hydrogen bonding along the TM3 helix is shown (*Right Inset*), highlighting the inward-facing S193 sidechain that caps the TM3 helix by hydrogen bonding with the backbone amide of the Y195 residue. (*B*) Exemplary current traces (TEVC, *X. laevis*) of THIK1 TM2/3 GS loop replacement constructs with the structured loop domain intact (TM2/3 GS^+^) or removed (TM2/TM3 GS^−^), showing basal currents (black) and response to application of 2 mM isoflurane (blue). (*C*) Current at 50 mV (TEVC, *X. laevis*) and (*D*) Isoflurane inhibition (%) of THIK1 WT and mutant channels. Mutants that show statistically significant changes in currents are highlighted in color. Values and statistics are listed in *SI Appendix,* Tables S1 and S2 and error bars represent mean ± SEM.

The outermost edge of the isoflurane binding pocket identified in our molecular dynamics simulations and functional screening is composed of residues that lie between the TM2 and TM3 helices. Unlike most other K2Ps, THIK channels contain an extended span of amino acids within their TM2/TM3 loop (*SI Appendix,* Fig. S6*A*) and in the THIK1 cryo-EM map resolved in this study, the distal section of this loop is ordered. A five-residue sequence, from Gly189 to Ser193, crosses the distal TM2 helix and adopts an inward-facing orientation previously unobserved in other K2P structures (Fig. 5*A* and *SI Appendix,* Figs. S4*D* and S6*B*).

As residues from the THIK1 TM2/TM3 loop contribute to the identified THIK1 isoflurane binding site and appear to modulate channel function, we examined the importance of the extended THIK1 TM2/TM3 loop for channel gating. We found that replacing the entire THIK1 TM2/TM3 loop region with a flexible glycine/serine repeat sequence (from residues Arg166 to Pro192, THIK1_TM2/3_GS−_) resulted in channels that exhibit enhanced current density and diminished sensitivity to the VA isoflurane (Fig. 5 *B*–*D*). The ordered region of the loop in our cryo-EM structure appeared to be the critical section responsible for these changes in THIK1 function, as replacement of only the unstructured portion of the TM2/TM3 loop (from residues Leu171 to Glu183, THIK1_TM2/3_GS+_) or deletion of this unstructured region (from Leu171 to Glu183, THIK1_TM2/3_∆_) had no impact on THIK1 basal currents or isoflurane sensitivity (Fig. 5 *B*–*D* and *SI Appendix,* Fig. S6 *C–E*). Meanwhile, mutations to key individual residues within the transition between the loop and TM3 recapitulate the reductions in VA sensitivity in the THIK1_TM2/3_GS−_ channels. These include the Trp190 and Lys191 residues that pack against the TM2 helix and the Pro192 residue that marks the end of the loop and the transition into the TM3 helix (Fig. 5 *A*, *C*, and *D* and *SI Appendix,* Fig. S6 *D–F*). The presence of a proline at the transition between the helical TM3 and the ordered region of the TM2/TM3 loop appears to be of particular importance (Fig. 5*A* and *SI Appendix,* Fig. S6 *D–F*), as multiple alternative substitutions at this position led to increased THIK1 basal currents and reduced VA sensitivity. The unique restraints on backbone dihedral angles inherent to proline permit the sidechain of the neighboring Ser193 to reorient inward toward the center of the TM3 helix and hydrogen bond with the backbone carbonyl of Tyr195 (Fig. 5*A*), providing an energetically favorable cap to the TM3 helix and transition into the structured loop domain. A THIK1 S193A mutant designed to disrupt this stabilizing hydrogen bond fully mimicked the functional impact of the THIK1_TM2/3_GS−_ construct in which the entire TM2/TM3 loop is eliminated (Fig. 5 *B* and *D* and *SI Appendix,* Fig. S6 *D–F*). These functional consequences of targeted disruptions to the stability of the ordered TM2/TM3 loop indicate the importance of this unique feature of the THIK1 structure, serving a regulatory role to inhibit THIK1 channel opening. The inclusion of key residues from this unique channel regulatory domain in the isoflurane binding pocket suggests a plausible mechanistic basis for the inhibitory effect of anesthetic binding on THIK1 channel function.

### A Lipid Bound behind the THIK1 Selectivity Filter.

In the THIK1 cryo-EM density map, we find a curved lipid-like density buried within the core of the THIK1 protein, directly behind the selectivity filter (Fig. 1 *A*–*C* and *SI Appendix,* Fig. S3*C*). This density fills a pocket between TM4 and the pore helices, at a site analogous to the previously described TREK1 cryptic modulatory binding site (33). In TREK1, this pocket is the binding site for small molecule activators ML335 and ML402 (33), contains well-characterized TREK1 W275S and G137I gain-of-function mutations (26, 34, 35), and is a target for modulatory anionic phospholipids (27). Point mutations and small molecules that target the TREK cryptic pocket have been shown to directly modulate the TREK1 selectivity filter (36, 37), raising the possibility that the observed lipid in THIK1 might similarly impact THIK1 selectivity filter function.

To test whether disruption of the lipid-bound pocket influences THIK1 function, we inserted mutations at residues surrounding the lipid density in the THIK1 structure and evaluated the function of the resultant channels. These include mutations to Arg92 and Arg258, a pair of residues poised to coordinate a bound lipid headgroup at the extracellular face of the pocket and mutations to THIK1 Phe262 and Gly105, positions that align to the TREK1 cryptic pocket W275 and G137 residues that result in large gain of function in TREK1 when mutated. While alanine substitution at the extracellular arginines had no impact on THIK1 function, both the G105I and F262S mutation markedly altered THIK1 basal currents (*SI Appendix,* Fig. S3*A*). While the F262S mutation reduced THIK1 currents and the G105I mutation enhanced them, in both cases, isoflurane inhibition remained unperturbed (*SI Appendix,* Fig. S3*B*). This retained isoflurane sensitivity in the functionally impactful lipid pocket mutants suggests that the lipid binding site in THIK1 is unlikely to be a target for anesthetic modulation and that this lipid site is likely to modulate THIK1 function through a distinct pathway that is independent of the mechanism by which VAs mediate Y-gate closure.

## Discussion

Despite considerable work determining the structure, gating, and pharmacology of numerous other K2P channel family members (38, 39), THIK channels have remained largely unexplored. Interest in THIK1 has intensified recently with the identification of its importance in regulating microglial function (3, 5) and the suggestion that THIK specific pharmacology might be used to modulate microglia in vivo (2, 14). In solving the cryo-EM structure of THIK1, we here identify unique structural features that define the THIK channel architecture. We utilize functional studies of anesthetic inhibition to probe THIK channel gating and pair this with anesthetic photolabeling to identify THIK1 VA binding sites. With this approach, we establish critical regions within the channel structure that modulate function and propose a speculative model for THIK1 gating (*SI Appendix,* Fig. S8) that accounts for the inhibitory effect of VA drugs and anticipates structural rearrangements that may underlie channel opening.

In nearly all K2Ps, conformational rearrangements of the TM4 helix play a central role in channel gating. For THIK1, the TM4 contributes the critical Y273 residue that constitutes the central pore gate. Inward-facing Y273 residues occlude the pore in the closed THIK1 structure, such that a conformational rearrangement of the TM4s is likely to be required for the THIK1 pore to open. The polar Y273 residues that form the Y-gate are positioned immediately below the selectivity filter, raising the possibility that in a conductive conformation these gate-forming tyrosine residues may directly contribute to dehydrating or coordinating potassium ions as they pass through the channel. Additional energetic barriers posed by involvement of the Y-gate tyrosine residues in ion permeation could provide a structural basis for the notably small single-channel conductance observed in electrophysiological recordings of THIK1 channels (12). Consistent with this notion, we observe EM density between the S4 selectivity filter Thr110 and Thr237 residues and the Y-gate in our cryo-EM map of THIK1 (*SI Appendix,* Fig. S4*A*). This “S5” site density is equivalently spaced and linearly aligned with the S1 to S4 potassium ion binding sites, and we therefore modeled this density as a potassium ion. The Y-gate Tyr273 hydroxyls lie below this ion and are rotated 45° along the pore axis relative to the S4 threonines, mirroring the square antiprism arrangement of potassium selectivity filter carbonyls at the S1 to S3 binding sites or the water hydration shell surrounding a pore vestibule bound potassium ion observed in prior structures of KcsA (40). While these observations suggest a role for the Tyr273 hydroxyls in shaping potassium ion permeation, we note that our THIK1 structure is in a presumed closed and nonconductive conformation, and we do not yet know how the Y-gate residues shift conformation upon THIK1 channel opening.

While it has long been evident that the TM2/TM3 loop in THIK channels contains an extended span of residues absent in most other K2Ps (*SI Appendix,* Fig. S6*A*), the functional relevance of this extended loop had not been previously explored. We find that a section of the THIK1 TM2/TM3 loop becomes ordered at the point when it crosses over the TM2 helix, allowing the loop to adopt a unique inward-facing orientation previously unobserved in other K2P channel structures. One clear consequence of this TM2/TM3 loop architecture is that the TM3s in THIK1 are drawn inward toward the central axis of the pore, bringing TM3 into close contact with the TM2 and TM4 helices (*SI Appendix,* Fig. S4*D*). The confluence of the THIK1 TM2, TM3, and TM4 helices at the cytoplasmic face of the channel forms the functionally critical isoflurane binding site we identify by photolabeling and molecular dynamics. The tight packing of the TM2 and TM3 helices against TM4 in the closed state structure of THIK1 appears poised to restrict TM4 movements that might allow for opening of the Y-gate.

Mutations to residues within the hydrophobic anesthetic binding pocket formed by the interface of TM2, TM3, and TM4 potentiate THIK1 basal currents and diminish isoflurane sensitivity. A similar pattern is observed for key residues that stabilize the ordered TM2/TM3 loop. As isoflurane inhibition of THIK1 ultimately modulates the central Y-gate, these functional results suggest allosteric communication between the region we identify as the functionally critical anesthetic binding site and the central TM4 Y273 pore gate. To explain the inhibitory effect of VAs on THIK1, we propose that binding of a lipophilic anesthetic to the hydrophobic interface formed by the TM2, TM3, and TM4 helices stabilizes the closed state conformation of the channel, allosterically biasing the transition between opening and closure of the Y-gate (*SI Appendix,* Fig. S8, *Right*). We anticipate that in a putative open state (*SI Appendix,* Fig. S8, *Left*) the ordered region of the TM2/TM3 loop may move outward away from the TM2 helix to adopt a more conventional outward-facing orientation. Such a rearrangement of the TM2/TM3 loop would be expected to alter the interface formed by the TM2, TM3, and TM4 helices at the anesthetic binding site, allowing for a conformational change in the position of the TM4 to permit opening of the central Y-gate. While “up/down” translational movements of the TM4 helix are critical for gating in mechanosensitive K2Ps, THIK1 gating may only require a more subtle repositioning of the TM4 helix, akin to those observed in K_ir_ channels where a simple rotation of the inner helices upon PIP2 binding leads to opening of the K_ir_ occlusive pore gate (41).

While THIK1 is inhibited by VAs, all other anesthetic-responsive K2Ps are potentiated by these agents, and we were surprised by the remarkable overlap of the THIK1 VA binding site with anesthetic modulatory sites in other K2Ps. In TREK and TASK channels, overlapping residues from the intracellular portions of the TM2, TM3, and TM4 helices mediate VA sensitivity (15–17, 42) and the THIK1 L146 VA binding site lies at a structurally equivalent site in the THIK K2P architecture. The L146 photolabeled site in THIK1 is located one helical turn away on TM2 from the azi-isoflurane binding site identified in the TREK1 K2P channel (27), and the residue that directly aligns to the TREK1 photolabeled site is the THIK1 Ile150. Among the THIK1 VA binding site mutants, Ile150 is the residue that exhibits the largest decrease in VA sensitivity (Fig. 4*E*) and is positioned directly at the center of the hydrophobic pocket that forms the THIK1 VA binding site.

In TREK channels, anesthetic binding appears to modulate the position of the TM4 gating helix to favor the highly active TM4 “up” conformation (17, 27). In TASK, residues involved in VA responsiveness (24, 42) and stereoselectivity (42) are located near the TM4 X-gate that seals the closed TASK pore (16, 24, 43), such that anesthetic potentiation is expected to promote X-gate opening. In our work, we find that VA binding promotes closure of the THIK1 TM4 Y-gate. In all cases, anesthetic binding appears to alter the dynamics of the TM4 helix to modulate channel activity. Despite the differing outcomes of anesthetic binding which are largely reflective of a diversity of gating mechanisms across the K2P superfamily, the site of action for VAs remains preserved. While certain VA agents have been shown to modulate mechanosensitive K2P channel function via an indirect effect on lipid bilayer organization (44), the extensive overlap between identified VA modulatory sites across TREK, TASK, and THIK K2P subtypes suggest that a direct mechanism of action via protein binding plays a critical role in the effects of clinically useful fluorinated ethers like isoflurane.

While the Y-gate serves to modulate the THIK1 pore below the selectivity filter, the presence of a lipid-like density buried behind the selectivity filter suggests that lipids may directly modulate THIK1 function. The lipid-bound pocket in THIK1 corresponds to an anionic phospholipid targeted site in the TREK1 channel (27, 33) and modulatory lipids have been found to bind to analogous sites in the KcsA (45) and KCNQ (46) potassium channels. Fatty acid signaling molecules are known to modulate THIK1 function in heterologous systems (1, 12, 13) and genetic variants in microglial lipid metabolic enzymes are known risk factors for development of neurodegenerative disease (47), suggesting that lipid composition in the microglial membrane could serve to modulate THIK1 function in vivo. While mutations introduced into the lipid binding pocket confirm the functional relevance of this site (*SI Appendix,* Fig. S3*A*), we have not directly examined the function of the purified C-terminally truncated THIK1 protein or the lipid dependence of the reconstituted THIK1 channel. As lipids were not added to the THIK1 purification, it is difficult to definitively assign an exact identity to the lipid-like density in our cryo-EM structure, though it must be either a copurified lipid from the mammalian cell membrane or a detergent molecule from the purification process. The removal of the balanced milieu of membrane lipids surrounding the THIK1 channel during detergent solubilization of the channel protein is one possible factor that could explain the clear discordance between the potentiated currents observed after C-terminal truncation of THIK1 (Fig. 1*D*) and the closed state conformation we observe in the cryo-EM structure. While a more complete accounting of the impact of lipids on THIK1 gating will require additional studies, these early glimpses into the structure, gating, and modulation of THIK1 channels open avenues to explore the roles of THIK1 channels in modulating microglial cell function and should accelerate development of specific pharmacological tools to modulate THIK1 channels in vivo.

## Methods

### Expression of THIK1 Channel Protein in Mammalian Cells.

To facilitate structural and photolabeling studies, we expressed and purified human THIK1 protein in HEK293 mammalian cells using a well-characterized baculovirus-based system (48). A C-terminally truncated isoform of the human THIK1 gene (Genbank ORF: NM_022054) bearing residues 1 to 309 of the full-length THIK1 sequence (referred to as hTHIK1_EM_ throughout the manuscript) was cloned into a pEG bacMam vector. GFP and HIS_10_ tags, followed by a PreScission Protease 3C cleavage site (LEVLFQ/GP), were added to the N terminus of the THIK1 gene to facilitate expression and purification, yielding the GFP-HIS-3C-hTHIK1∆309 construct used to produce baculovirus. This construct was transformed into DH10Bac *Escherichia coli* cells to produce and isolate Bacmid DNA.

To produce initial P1 baculovirus, SF9 insect cells (adherent culture in 6-well plates, 8 × 10^5^ cells/mL) were transfected with 2 µg of Bacmid DNA using a Cellfectin II transfection reagent. Seven days after transfection, evidence of successful viral production and infection was evident by a GFP signal on the SF9 cell membranes and P1 virus was collected by harvesting transfected cells, disruption of SF9 cell membranes by trituration in a 5 mL syringe, and subsequent low-speed centrifugation at 4,000 rpm for 10 min. Following centrifugation, the supernatant containing the P1 virus was supplemented with 2% fetal bovine serum (FBS) and sterile filtered (0.22 µm) to prevent bacterial contamination. To further amplify the baculovirus for large-scale protein expression, 2 more rounds of infection (P2 & P3) were performed using larger volumes of SF9 cells in suspension culture. SF9 cells were grown in suspension at 28 °C, shaken at 125 rpm, and maintained at 75% humidity. For P2 virus production, 2 × 10^6^ cells/mL were seeded with a 1:1,000 dilution of the P1 virus stock and harvested after 5 d of incubation. For P3 virus production, 3 × 10^6^ cells/mL were seeded with a 1:20,000 dilution of the P2 virus and harvested after 4 d.

For large-scale production of THIK1 protein in mammalian cells, the P3 virus was used to infect a suspension culture of HEK293 GnTi^−^ cells, grown to a density of 2.4 − 2.8 × 10^6^ cells/mL and infected with a 10% v/v ratio of P3 virus to HEK293 cell culture. The infected HEK293 GnTi^−^ cultures were incubated at 37 °C, 8% CO_2_, 75% humidity and shaken at 125 rpm for 12 h, followed by addition of 10 mM sodium butyrate and grown for an additional 24 h at 30 °C. At the 36 h timepoint, 50 to 90% of the cells were GFP fluorescent and were harvested by centrifugation at 4,500 rpm for 20 min. The cell pellet was then flash frozen in liquid nitrogen and stored at −80 °C.

### THIK1 Protein Purification.

The frozen HEK293 GnTi^−^ cell pellet was resuspended in 5 mL of a 30% glycerol solution and then lysed for 25 min at 4 °C in 200 mL of a hypotonic solution [20 mM KCl, 0.5 mM MgCl_2_, 2 mM DTT, 10 mM Tris pH 8] supplemented with 0.2 mg/mL DNase, 2 tablets EDTA-free Complete Protease inhibitor cocktail (Roche), and 1 mM phenylmethylsulfonyl fluoride. Total cell membranes were collected by ultracentrifugation at 100,000 × g for 40 min, followed by mechanical homogenization of the membrane containing pellet in extraction buffer [20 mM Tris pH 8, 10 mM LMNG, 2 mM CHS, 300 mM KCl, 2 mM DTT, 1 mM PMSF, 1 Protease inhibitor cocktail tablet, and 0.2 mg/mL DNase]. This sample was then rotated at 4 °C for 2 h to solubilize the cell membranes, followed by centrifugation at 35,000 × g for 45 min at 4 °C to remove any remaining cellular debris. 3 mL of sepharose resin coupled to homemade GFP nanobodies (49, 50) was then added to the solubilized membranes and incubated overnight on an orbital rotor at 4 °C. The THIK1 bound GFP resin was then collected on a column and washed with 10 column volumes of glyco-diosgenin (GDN) containing buffer [0.05% GDN, 20 mM Tris pH 8, 300 mM KCl, 2 mM DTT, 1 Protease inhibitor cocktail tablet] to remove nonspecifically bound protein.

To release the immobilized N-terminally GFP tagged THIK1 protein from the GFP-nanobody resin, home purified PreScission protease (3C) was added to the column and incubated overnight at 4 °C. The THIK1 protein was then eluted from the GFP-nanobody resin, concentrated in a 50 kDa molecular weight cut-off (MWCO) Amicon Ultra Centrifugal Filter (Millipore), and run on a Superdex 200 Increase 10/300 GL column equilibrated in 150 mM KCl, 20 mM Tris pH 8, and 0.05% GDN. The purified THIK1 protein was concentrated (50 kDa MWCO) to 2.4 mg/mL and analyzed for purity by SDS-PAGE [12% (wt/vol) gels; Bio-Rad], followed by staining with Coomassie blue. In the final cryo-EM sample used for Krios data collection, a repeat size exclusion chromatography run at a lower GDN concentration (150 mM KCl, 20 mM Tris pH 8, and 0.01% GDN) was performed. During sample screening, we found that lowering the detergent concentration improved micrograph quality, most notably by increasing THIK1 particle distribution in regions of high-contrast thin ice.

### Cryo-EM Data Collection, Processing, and Model Building.

Purified THIK1 protein was frozen on R1.2/1.3 UltraAUfoil 300 mesh grids (Quantifoil) that were glow discharged for 80 s at −25 mA using a Pelco easiGlow glow discharge system (Ted Pella). A 3 µL volume of 2.4 mg/mL purified THIK1 protein was added to the grids and freezing was carried out using a Vitrobot Mark IV (FEI). Samples were equilibrated in the Vitrobot chamber for 20 s at 100% humidity and 21 °C, blotted for 3 s with a blot force of +5, and then plunge frozen in liquid ethane.

The complete THIK1 dataset is composed of micrographs from two cryo-EM grids that contain identical THIK1 sample and were acquired in two separate sessions on the same microscope, a 300 kV Titan Krios G3i equipped with a Gatan K3 imaging system and a GIF quantum energy filter set to a slit width of 20 eV. A total of 7,299 micrographs from grid A and 6,947 micrographs from grid B were collected, using Leginon v 3.6 software (51). For both datasets, intermediate frames were collected every 0.05 s for a total of 40 frames per micrograph. The total exposure for both grids was 1.80 s, at a dose rate of 26.63 e^−^/Å^2^/s for grid A and 26.96 e^−^/Å^2^/s for grid B, resulting in accumulated doses of 47.94 e^−^/Å^2^ for grid A and 48.54 e^−^/Å^2^ for grid B. Both datasets were collected at 105,000× nominal magnification, with a calibrated pixel size of 0.4125 Å/pixel used for data processing.

All data processing was performed in RELION 3.1.2 (52) and details of the data processing scheme and final processing results are described in *SI Appendix,* Figs. S1 and S2. Briefly, dose fractionated images were 3× Fourier binned, gain normalized, dose-weighted, and motion corrected in 10 × 10 patches using RELION’s implementation of Motion-Cor2-1.4.0 (53). CTFFIND4.1 (54) was used to perform contrast transfer function (CTF) and defocus estimation. Particle picking was performed in crYOLO (55) (version 1.7.6), after training the generic crYOLO model on a subset of our motion corrected micrographs. Particle pick locations from crYOLO were transferred back into RELION and used for particle extraction, using a 196-pixel box size. For each of the datasets, 3 rounds of 2D classification were performed in parallel and the particles that comprised the final 2D classes selected from Grid A were used as input for ab initio model generation. This ab initio 3D model was then used as input for the first of two rounds of 3D classification performed in parallel on each of the two datasets. All 3D classes from both datasets that contained strong transmembrane domain density were pooled together for subsequent refinement. From this point onward, both datasets were joined and refined together, using the best 3D class from Grid B (low pass filtered to 20 A) as the first initial model for refinement. To improve 3D reconstruction, iterative rounds of beam tilt estimation, anisotropic magnification estimation, per-particle CTF estimation, and particle polishing were performed, until 3D refinements converged and no longer improved map quality or estimated resolution. Remaining low contrast or poorly aligned particles were removed by additional rounds of 2D and 3D classifications, eliminating classes that lacked strong transmembrane domain signal relative to the detergent micelle density. Application of C2 symmetry improved resolution estimates at intermediate points during the processing pipeline but ultimately led to detrimental averaging of the lower-resolution regions in the TM2/TM3 loop and was abandoned in later reconstructions. At later stages of the processing pipeline, refinement with SIDESPLITTER (56) helped diminish local overfitting and resulted in improvements in map quality and estimated resolution. The final particle stack was ultimately resampled from the 3× Fourier binned pixel size of 1.24 Å/pixel to a smaller pixel size of 0.79 Å/pixel, by re-extraction during RELION particle polishing. At this new smaller pixel size, subsequent refinements resulted in lower-resolution estimates but nonetheless showed improvements in map quality. Reported resolution estimates reflect an FSC curve cut-off of 0.143 and were determined during RELION postprocessing, performed with a volume mask designed to exclude the detergent micelle surrounding the THIK1 protein. Local resolution estimates were calculated in RELION.

To facilitate model building and data representation, two map sharpening tools were used. After the final refinement in RELION, unmasked and unfiltered half-map reconstructions were sharpened using DeepEMhancer (57), while the final full refinement reconstruction was sharpened with EMReady (58). The THIK1 structure was ultimately built using a combination of the final postprocess map from RELION and these sharpened maps. As a starting point for model building, two copies of the alphafold predicted structure of the human THIK1 monomer were docked into the sharpened THIK1dtS309 cryo-EM density map using UCSF chimera 1.16 (59). This model was then manually adjusted in COOT (60) (version 0.9.8.1) to fit into the cryo-EM density map. Following manual building, global real space refinement with stereochemistry restraints was performed in Phenix (61). Further round of manual inspection and correction of sidechain outliers in COOT and real space refinement in Phenix ultimately produced the final THIK1 model. The profile of the ion-conduction pathway was calculated using HOLE (62). For the CAP glycosylation sites, we modeled each sugar as a (GIcNAc)2 Mannose5, as the THIK1 channel protein was expressed and purified from HEK293F GNTI^−^ cells lacking the N-acetylglucosamine enzyme required for more complex glycosylation. The RELION postprocessed map used for PDB validation showed high-resolution data only for the initial N-Acetylglucosamine moiety and this is all that was built into the deposited model. In the unsharpened maps or DeepEMhancer processed maps, additional density for the sugar moieties is present and we modeled as much of the (GIcNAc)2 Mannose5 sugar as would fit within the experimental data, as shown in *SI Appendix,* Fig. S3*D*. We note that the relatively lower resolution in the cryo-EM map at these glycosylation sites is likely to reflect heterogeneity in the pose of each of the attached sugar moieties.

### Electrophysiological Recordings.

All electrophysiological recordings in this study were performed on *Xenopus laevis* oocytes, using the two-electrode voltage clamp (TEVC) technique. The full-length human THIK1 gene was cloned into a pFAW plasmid vector directly downstream from a T7 RNA polymerase promoter, to facilitate in vitro production of 5′ capped THIK1 RNA (cRNA) for injection into oocytes. All THIK1 point mutants described in the manuscript were created in this vector backbone, using site directed mutagenesis carried out with a Phusion High-Fidelity DNA polymerase kit (ThermoScientific). Truncations or insertions were created using sequence-based ligase independent cloning (SLIC) techniques. These include a THIK1 1-309 truncation that mirrors the cryo-EM structural construct (THIK1_EM_), THIK1 1-331 truncation that follows the known IETD caspase 8 cleavage site present at positions 327 to 330 (THIK1_CAS_), the TM2/TM3 loop deletion construct with removal of all residues between and including Leu171 to Glu183 (THIK1∆), or the TM2/TM3 loop GS replacement at either Arg166 to Pro192 (TM2/3 GS^−^) or Leu171 to Glu183 (TM2/3 GS^+^), where a repeating GGGS sequence was inserted to replace residues within the ranges noted.

In preparation for in vitro cRNA production, the pFAW vector was linearized using the FastDigest Mlu1 restriction enzyme (ThermoScientific). The linearized THIK1 containing pFAW plasmid DNA was purified using the QIAquick Nucleotide Removal Kit (Qiagen) and THIK1 cRNA was produced using an mMessage mMachine Kit (T7 promoter, Ambion, Life Technologies). Products of the in vitro cRNA reaction were treated with DNAse, purified with an RNeasy RNA cleanup kit (Qiagen) and stored at −80 °C prior to injection into oocytes. For all THIK1 WT and mutant recordings, 2.5 ng of cRNA was injected into defolliculated stage IV-V *Xenopus laevis* oocytes (Xenoocyte, Dexter, MI) and incubated in 1× Leibovitz’s L15 media (supplemented with 5 mM HEPES and 2.5% penicillin/streptomycin) at 16 °C prior to recording.

All TEVC recordings were performed 24 to 36 h after microinjection. Borosilicate microelectrodes were pulled to a resistance of 0.2 to 2.0 MΩ and backfilled with 3 M KCl solution. Unless otherwise noted, oocytes were perfused with ND96 solution (96 mM NaCl, 2 mM KCl, 1.8 mM CaCl_2_, 2.0 mM MgCl_2_, 5 mM HEPES; pH 7.4) at a rate of 2.5 mL/min. Currents were acquired using an OC-725C Oocyte Clamp amplifier (Warner Instruments), controlled by pClamp10 software (Molecular Devices). A Digidata 1332A digitizer (MDS Analytical Technologies) was used to digitize the signal at 2 kHz.

THIK1 IV curves were obtained from an 80 mV holding potential, using stepwise (+20 mV/step) rectangle pulses (500 ms) from −150 to +50 mV. Isoflurane inhibition experiments were carried out using a ramp protocol (−150 to +50 mV, 5 ms full sweep duration; 10 s wait time between each sweep). The temperature of the perfused solution was monitored using a CL-100-controlled thermistor placed in the bath solution immediately downstream of the oocyte and controlled by a SC-20 in-line heater/cooler combined with an LCS-1 liquid cooling system operated by the CL-100 bipolar temperature controller (Warner Instruments). If not stated otherwise, recordings were carried out at room temperature (21 °C). All TEVC data presented in the manuscript were obtained from at least two independent batches of ovaries, with a minimum of n = 5 experiments per condition.

For isoflurane experiments, a 99% isoflurane solution (Baxter Healthcare Corporation) was added to 100 mL of ND96, until a clear phase separation was achieved. The mixture was sealed tightly in a glass bottle and stirred vigorously overnight prior to usage. The maximal solubility of isoflurane in salt solution at room temperature has been empirically determined to be 15 mM (63) and 2 mM isoflurane working concentrations were achieved by appropriate dilution of the aqueous phase of the saturated solution into ND96, using sealed syringes to prevent environmental loss of anesthetic. For azi-isoflurane electrophysiology, the pure compound at an analytically determined concentration of 6.25 M was dissolved directly into ND96 to obtain a working concentration of 1.5 mM and was vortexed vigorously for 2 min prior to use.

### Azi-Isoflurane Photolabeling, Protein Digestion, and Mass Spectrometry Analysis.

For THIK1 photolabeling, 30 μM azi-isoflurane was added to a 1 mg/mL solution of purified hTHIK1_EM_ protein in 0.01% GND detergent. The sample was equilibrated on ice in the dark for 5 min before being exposed to a 300 nm RPR-3000 Rayonet lamp in a 1-mm path-length quartz cuvette through a WG295 295-nm glass filter (Newport Corp., Irvine, CA) for 25 min. For isoflurane protection studies, the equivalent photolabeling experiment was repeated in the presence of 3 mM isoflurane.

Following photolabeling, the THIK1 protein was run on an SDS-PAGE gel. The hTHIK1_EM_ band was excised, destained, dehydrated, and dried, followed by addition of 5 mM DTT and 50 mM NH_4_HCO_3_. Samples were then treated with iodoacetamide (IAA), followed by sequencing grade-modified trypsin at a 1:20 protease: protein ratio (w:w), with addition of 0.2% (w/v%) ProteaseMax™ surfactant. Proteins were digested overnight at 37 °C and then diluted with NH_4_HCO_3_ and 0.02% ProteaseMAX Surfactant, followed by addition of sequencing grade chymotrypsin to a final 1:20 protease:protein ratio (w:w). Proteins were again digested overnight at 37 °C and then acidified with acetic acid, followed by centrifugation to remove insoluble debris. Samples were then desalted using C18 stage tips, dried by speedvac, and resuspended in 0.1% formic acid prior to mass spectrometry analysis.

Mass spectrometry was performed as previously reported (17, 64). Briefly, desalted peptides were injected into a Thermo LTQ Orbitrap XL Mass Spectrometer (Thermo Fisher Scientific, Waltham, MA). Peptides were eluted over 100 min with linear gradients of acetonitrile in 0.1% formic acid in water. Spectral analysis was conducted using MaxQuant to search b and y ions against the hTHIK1 sequence. All analysis included dynamic oxidation of methionine (+15.9949 m/z) as well as static alkylation of cysteine (+57.0215 m/z; iodoacetamide alkylation). Filter parameters were Xcorr scores (+1 ion) 1.5, (+2 ion) 2.0, (+3 ion) 2.5, deltaCn 0.08, and peptide probability 0.05. Photolabeled peptides were identified by additional dynamic azi-isoflurane modification (+195.97143 m/z). The trypsin/chymotrypsin digests were searched without enzyme specification with a false discovery rate of 0.01.

### Molecular Dynamics Simulations.

All simulations were conducted using NAMD3 (65) with CHARMM36 force fields (66, 67), existing isoflurane parameters (68), and TIP3P water. Systems were prepared using the CHARMM-GUI tool (69, 70). As a region of the TM2/TM3 loop is unresolved in the THIK1 cryo-EM map, we took two alternative approaches to modeling this region of the THIK1 protein for the MD simulations. In one of the MD production simulations (L146_loop), the TM2/TM3 loop conformation was predicted using SuperLooper2 (71). In an independent run (L146_∆), the unstructured region of the TM2/TM3 loop between Arg166 and Leu187 was deleted from the THIK1 map, shortening the length of the TM2/TM3 loop. Production simulations were conducted in the isothermic–isobaric ensemble with Langevin thermostat. The lipid bilayer consisted of 70% 1-palmitoyl-2-oleoyl-sn-glycero-3-phosphocholine (POPC) and 30% cholesterol. The L146_loop production equilibration simulation was 87 ns in length and the L146_∆ simulation was 50 ns in length. In both cases, the isoflurane molecule equilibrated into a stable orientation.

The Streamlined Alchemical Free Energy Perturbation (SAFEP) methodology (72) was used to determine the absolute binding free energy of isoflurane. Briefly, SAFEP introduces a limited set of restraints on the ligand to maintain a bound conformation during alchemical transformation and preferentially samples states that most contribute to the binding free energy. The energetic cost of these restraints is then corrected to yield an accurate absolute binding free energy. Here, we used an upper bound for the distance from bound conformation restraint of 5 Å, and a volumetric restraint radius of 6 Å. The Bennett acceptance ratio method was used to calculate free energy differences. The initial pose used for free energy transformation was taken as the ending isoflurane configuration in the L146_loop simulation, while an intermediate state (at 67 ns simulation time) and ending configurations were both used for free energy transformation in the case of the L146_∆ simulation. Each free energy transformation was calculated as the sum of decoupling the unbound ligand from bulk water solvent phase to gas phase, recoupling the ligand from gas phase to the protein-bound state, and restraint corrections. The bulk water solvent phase calculation was split into 171 windows, and the recoupling to protein calculation was split into 120 windows. Each window was 2 ns in length. Integrated double-wide sampling was used to sample both forward and backward directions simultaneously. The restraint corrections were calculated using thermodynamic integration.

### Fluorescent Cell Imaging of THIK1.

For fluorescent cell imaging, 2 × 10^5^ HEK293S GnTi^−^ cells were seeded into 6-well plates containing 2 mL of DMEM (Gibco) supplemented with 10% FBS in each well. These plates were maintained in a 5% CO_2_ incubator at 37 °C until cells reached 60 to 70% confluency (~48 h). In preparation for transfection, each well was first washed with 1 mL DPBS and then incubated for 1 h at 37 °C with 2 mL Freestyle media (Gibco) containing 2% FBS. 1 µg of N-terminal GFP tagged THIK1 WT, N59Q, N65Q, or N59Q N65Q in a pEG vector was mixed with Lipofectamine (Invitrogen) transfection reagent and the mixture was added to the cells. Transfection was allowed to proceed for 8 h and was terminated by exchange into fresh Freestyle media. The following day, cells were imaged using an all-in-one inverted fluorescent microscope of the BZ-X series (Keyence) equipped with a cooled CDC camera. Images were taken using a plan apochromat 4× or 40× objective in brightfield or using a GFP filter (OP-87763), with an excitation wavelength of 470/40 nm and emission wavelength of 525/50 nm. After cell imaging, the transfection efficiency (% of transfected cells) for each construct was determined using a GFP filter equipped Nexcelom cell counter (Revvity). Brightness and contrast of fluorescent images were adjusted for visualization using Inkscape 1.0.1.

## Supplementary Material

Appendix 01 (PDF)

## Data Availability

The THIK1 cryo-EM density map and associated molecular model are deposited in the Electron Microscopy Data Bank (http://www.ebi.ac.uk/pdbe/emdb/) and Protein Data Bank (http://www.rcsb.org) with accession codes EMD-47258 (73) and PDB-9DWN (74).
