# Supplementary material for: The cryo-EM structure and physical basis for anesthetic inhibition of the THIK1 K2P channel
Source: Proc Natl Acad Sci U S A. 2025 Apr 3;122(14):e2421654122. doi: 10.1073/pnas.2421654122 (PMC12002230; doi:10.1073/pnas.2421654122)
Supplement: Supplementary file 1 — Appendix 01 (PDF) [file pnas.2421654122.sapp.pdf]

Supporting Information for
The cryo-EM structure and physical basis for anesthetic inhibition of the THIK1 K2P channel.

Elena B. Riel, Weiming Bu, Thomas T. Joseph, Leila Khajoueinejad, Roderic G. Eckenhoff, Paul M. Riegelhaupt

Corresponding author: Paul M. Riegelhaupt
Email: par9082@med.cornell.edu

This PDF file includes:

Figures S1 to S11
Tables S1 to S3

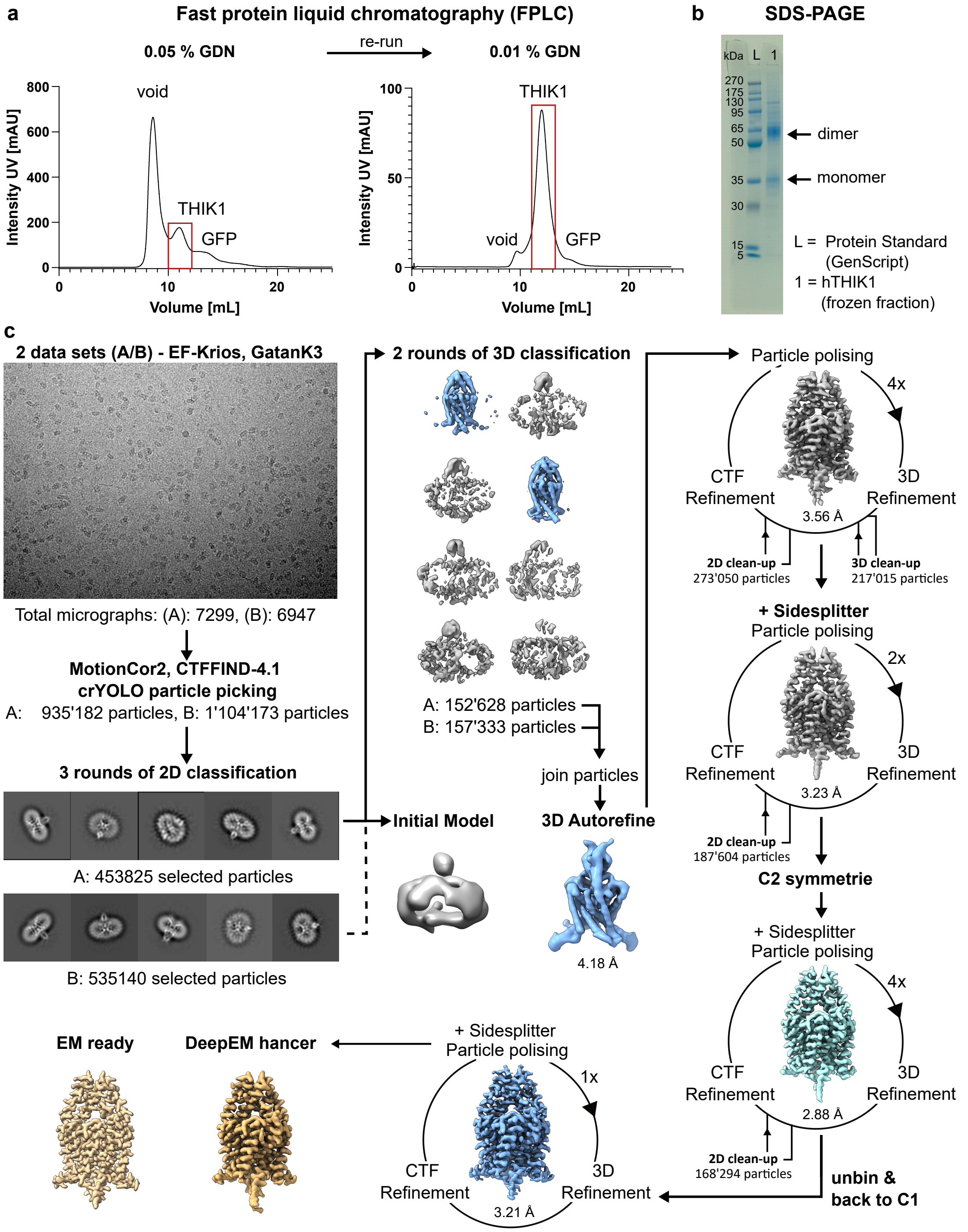

Fig. S1. – Protein Purification and Cryo-EM processing scheme. a Fast protein liquid chromatography profile of the THIK1 sample used to prepare cryo-EM grids. **b** SDS page of the collected THIK1$_{EM}$ fraction. **c** Cryo-EM processing scheme from two data sets using Relion, crYOLO, Sidesplitter, DeepEMhancer and EMready.

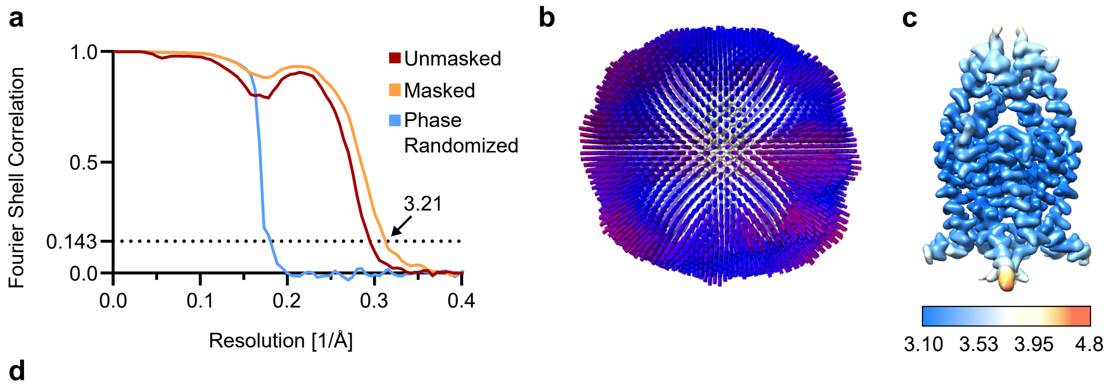

hTHIK1 model / map validation				
PDB	9DWN		**Model composition**	
EMDB	EMD-47258		Nonhydrogen atoms	4323
**Data collection**	**Collection A**	**Collection B**	Protein residues	540
Magnification	105,000X	105,000X	Ligands K / NAG / water	5/4/2
Voltage (kV) 300	300	300	**B-factor**	
Electron exposure (e-/Å2)	47.94	48.54	Protein	10.20/115.45/41.61
Defocus range (µm)	0.5 – 2.5	0.5 – 2.6	Ligands	11.34/107.15/88.37
Pixel size (Å)	0.4125	0.4125	**RMSD**	
**Processing**			Bond length (Å)	0.003
Final symmetry imposed	C1		Bond angle (°)	0.569
Initial particle images (no. Post 2D)	453825	535140	**Validation**	
Final particle images (no.)	168294		Molprobity score	1.38
Map resolution (Å) at FSC 0.143 (Relion)	3.21		Clash score	5.82
Map local resolution range (Å)	3.1 - 4.8		Poor rotamers (%)	0
**Refinement**			Ramachandran plot	
Initial model used	AlphaFold: AF-Q9HB14-F1		Outliers (%)	0
FSC model (0/0.143/0.5) (Phenix)	2.6/2.8/3.3		Allowed (%)	2.26
Map sharpening b-factor (Å2)	-132.343		Favored (%)	97.74

Fig. S2. THIK1 Cryo-EM map validation. **a** Fourier Shell Correlation (FSC) curves, **b** angular distribution, and **c** local resolution estimates, 3.1 (blue) - 4.6 Å (red). **d** THIK1 Cryo-EM dataset validation data table. Model validation performed in Phenix.

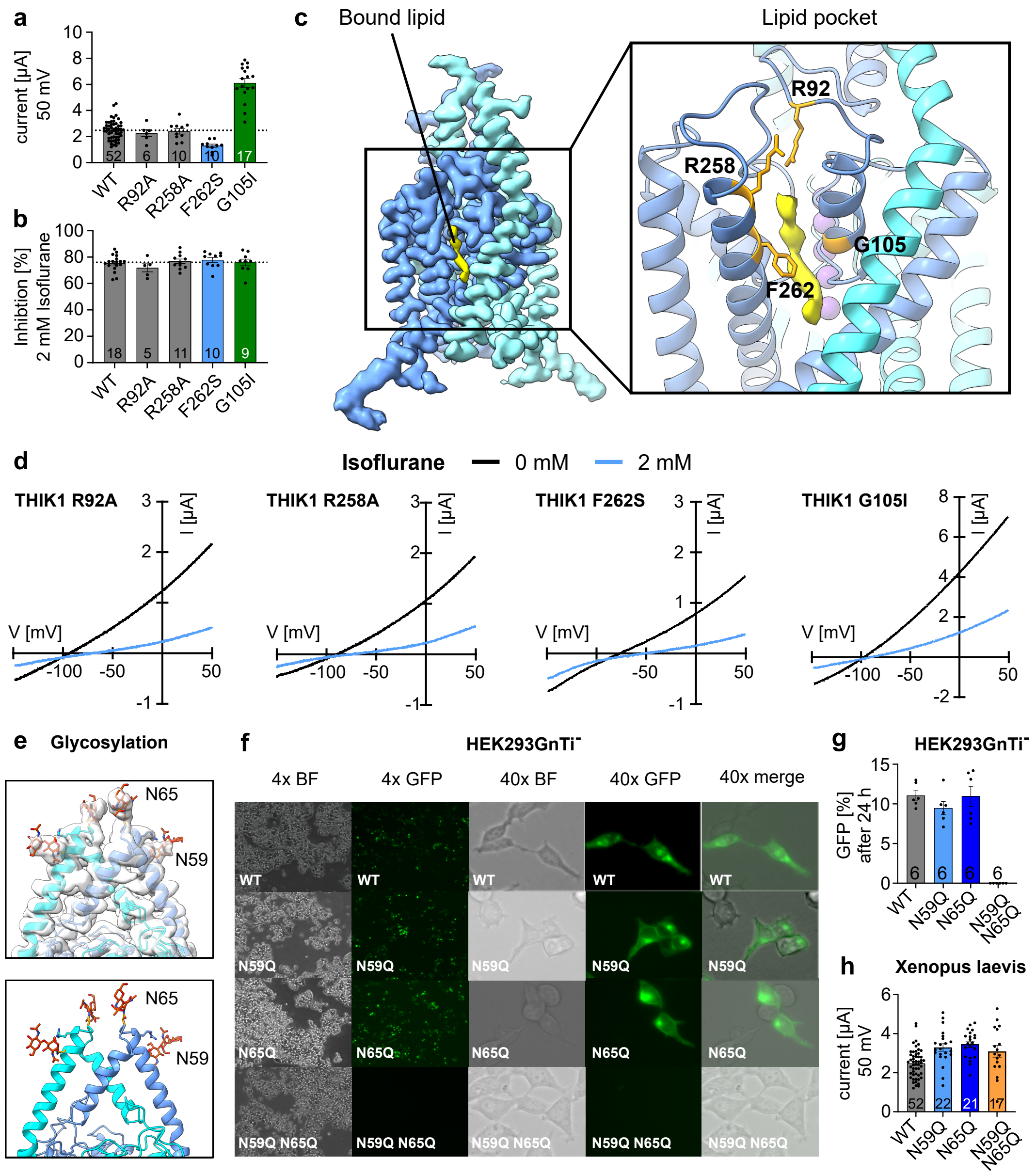

Fig. S3. THIK1 glycosylation and lipid occupancy. **a** Current at 50 mV (TEVC, *Xenopus laevis*) and **b** Isoflurane inhibition [%] of THIK1 WT (black) or lipid pocket mutants. **c** Cryo-EM map and THIK1 structural model depicting the bound lipid density (yellow) within a pocket behind the selectivity filter of the THIK1 channel, highlighting the positions of lipid binding site residues examined by electrophysiology. **d** Exemplary current traces of THIK1 lipid pocket mutants, prior to (black) and after (blue) application of 2 mM Isoflurane. **e** DeepEMhancer wideTarget cryo-EM map (above) and structural model (below) of THIK1, depicting additional density at the glycosylation sites N59 and N65 and (GlcNAc)2 Mannose5 sugars modelled within the density **f** Fluorescence microscopy images of HEK293 GnTi- cells transfected with N-terminally GFP tagged THIK1 WT, N59Q, N65Q, or a N59Q N56Q double mutant construct using a 4x or 40x magnification, in bright field (BF) or with a GFP filter. **g** GFP positive cells [%] after 24 h expression time. **h** Current at 50 mV (TEVC, *Xenopus laevis*) from THIK1 WT, N59Q, N65Q or N59Q N65Q double mutant. For all panels, values and statistics are listed in Table S1 & S2 and error bars represent mean ± SEM.

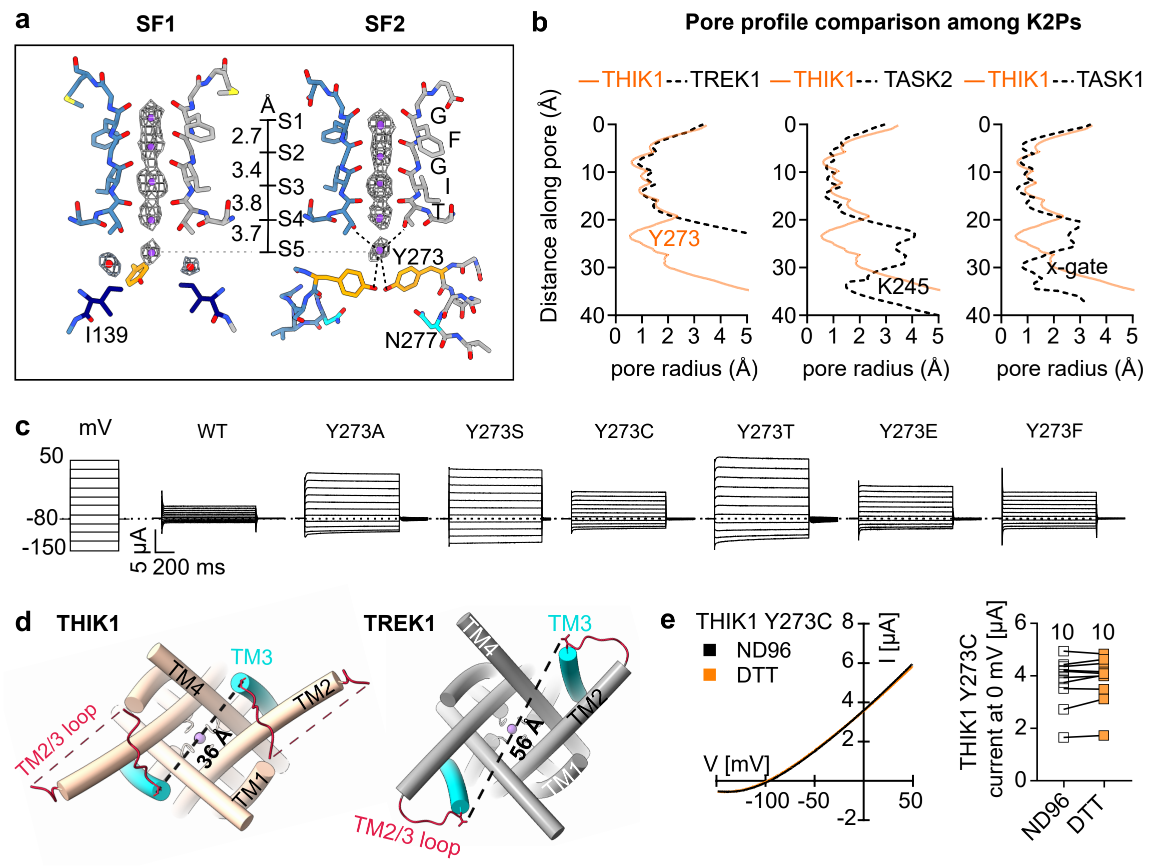

Fig. S4. THIK1 pore architecture. a Structure of the THIK1 selectivity filter (SF) showing four potassium ions (purple) occupying the S1-S4 ion coordination sites. An additional S5 site ion, coordinated by SF residue Thr119 and Thr237, the intracellular gate residue Y273 (orange), and two structures water molecules (red) within the cavity between the Y-gate and the selectivity filter. Ions and waters were modelled into volume densities (shown as grey mesh) obtained from the final postprocess THIK1 Cryo-EM map at a visualization threshold of 0.024. Spacing in angstroms along the pore axis for each of the S1-S5 ions is shown. Residues that impact Y-gate closure are shown, including I139 (dark blue) and N277 (light blue). **b** Pore radius profiles determined by HOLE, comparing the pore of THIK1 obtained in this study (orange) with prior models (black dashes lines) obtained for TREK1 (PDB: 8DE9), TASK2 (PDB: 6WLV) and TASK1 (PDB: 6RV3). **c** Exemplary traces (TEVC, Xenopus *laevis*) of THIK1 WT or Y-gate mutant channels measured via step protocol (-150 to 50 mV, 20 mV steps). **d** Cytoplasmic view of the THIK1 (left) or TREK1 (right, PDB: 8DE9) channel, highlighting the unobstructed pores in both channel types and the inward vs. outward positioning of the TM2/TM3 loop structures, with resultant differences in the distance between the ends of the TM3 helices (teal) for THIK1 (at THIK1 Ser193) versus TREK1 (at TREK1 Thr204). **e** Exemplary traces (TEVC, *Xenopus laevis*) of THIK1 Y273C mutant channels measured in ramp protocols (-50 to +150 mV) before (black) and after (orange) application of 10 mM DTT for 5 min. Current densities of Y273C measurements (as above) before and after DTT application.

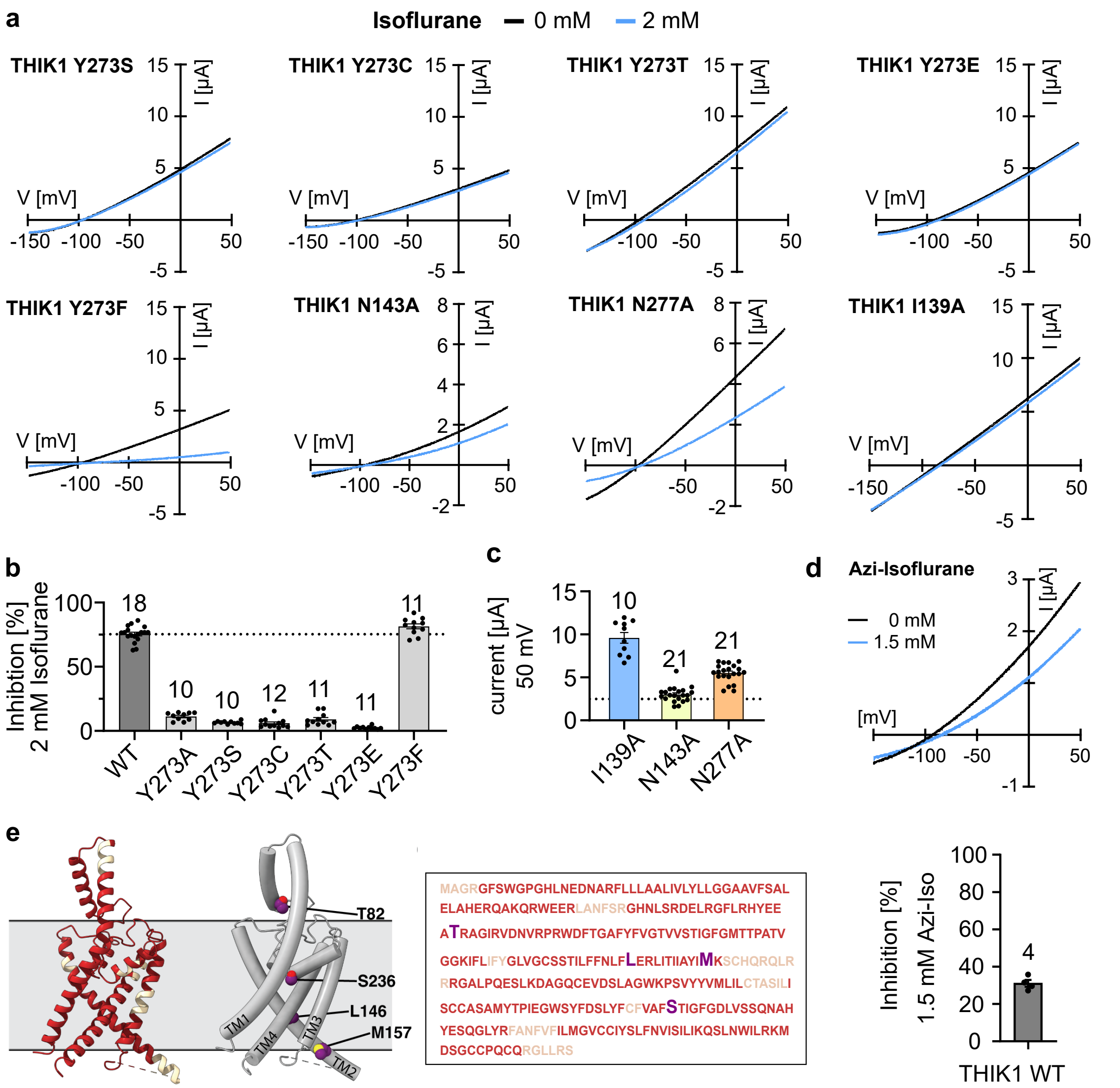

Fig. S5. Functional and photolabeling studies of THIK1 isoflurane inhibition a. Exemplary traces (TEVC, Xenopus laevis) of the THIK1 pore gate mutants Y273 and I139, and the N-ring asparagine residues N143 and N277, measured in ramp protocols (-50 to +150 mV) before (black) and after (blue) application of 2 mM Isoflurane. **b** Isoflurane inhibition % of THIK1 WT and Y273 mutants after treatment with 2mM Isoflurane. **c** Basal current at 50 mV for I139A, N143A, and N277A THIK1 mutant channels. The mean level of THIK1 WT current at 50 mV is represented by the dotted line **d** Exemplary trace (TEVC, Xenopus laevis) of the response of THIK1 WT to application of 1.5mM Azi-isoflurane (above) and azi-isoflurane inhibition % of THIK1 WT (below) **e** Photolabeling coverage mapped on the THIK1 monomer structure, colored by (left) regions positively identified in mass spectrometry experiments (red) and missing coverage (beige) or by azi-isoflurane photolabeled sites (right, dark blue). MS results are also mapped on the THIK1 protein sequence, shown in the inset box. For all panels, values and statistics are listed in Table S1 & S2 and error bars represent mean ± SEM

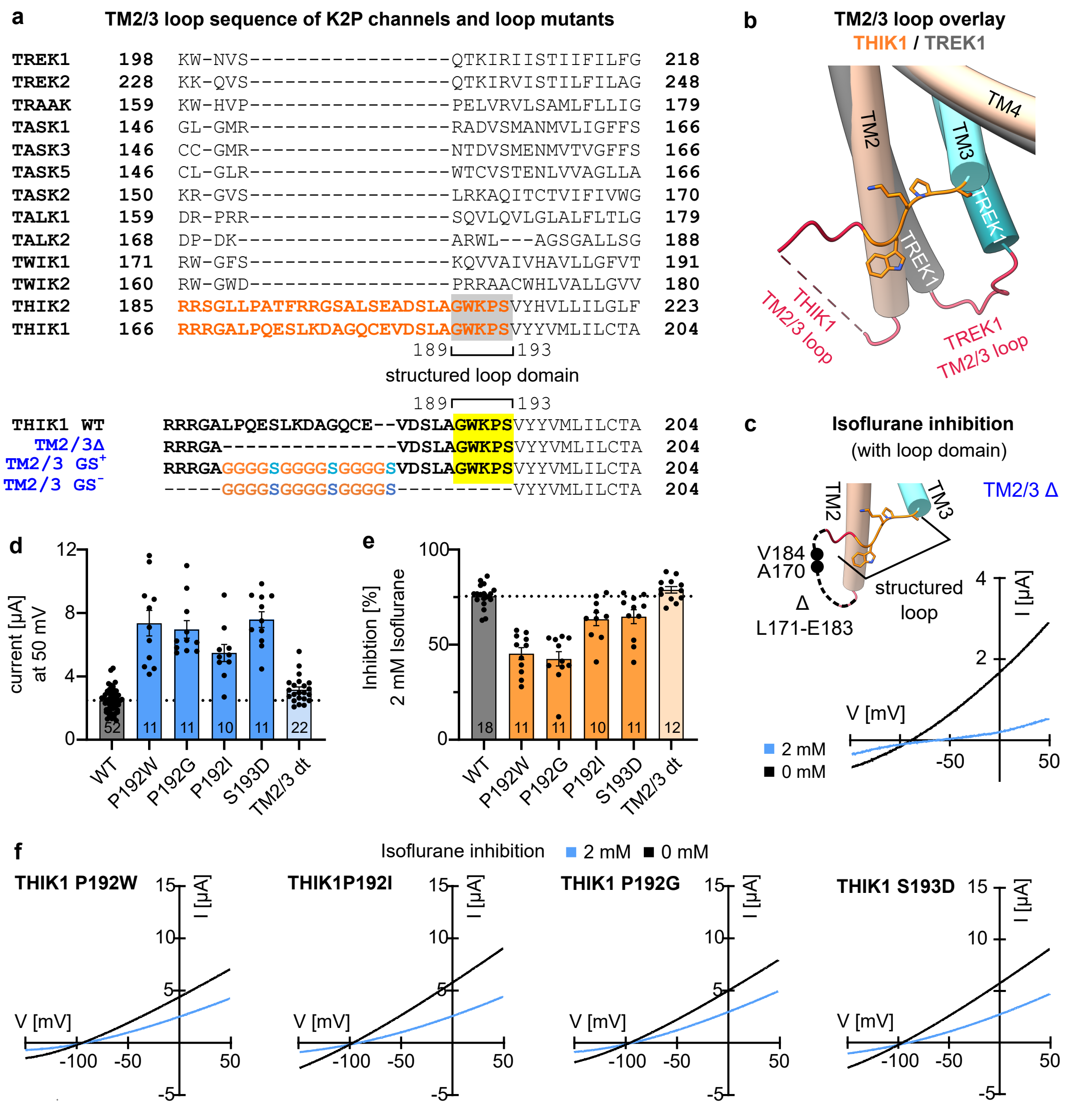

Fig S6. Isofluran sensitivity of the structured M2/3 loop domain mutants. **a** K2P sequence alignment of the M2/M3 loop region, showing the unique loop extension present in THIK subfamily members (orange), with the structured region within the loop (GWKPS) noted. The TRESK K2P channel is omitted in this alignment, as TRESK is known to contain a large calcineurin binding domain inserted between TM2 and TM3. Alignment of the THIK1 WT amino acid sequence with the TM2/3 loop mutant constructs, including the TM2/3 Δ construct in which the structured loop domain is left intact and the unstructured residues from Leu171 to Glu184 are deleted, the TM2/3 GS^- construct in which the structured loop domain is left intact and the unstructured section of the loop is replaced by a GS linker, and the TM2/3 GS^+ construct in which the entire TM2/TM3 loop is replaced by a GS linker. **b** Zoomed in structure overlay of the THIK1 and TREK1 (PDB: 8DE8) M2/M3 loop region, showing the short loop between TM2/TM3 in the TREK1 structure and the notably extended and partially structured THIK1 loop. **c** Cartoon of the THIK1 TM2/3 Δ mutant construct and exemplary current traces acquired prior to (black) and after (blue) treatment with 2 mM Isoflurane. **d** Basal current at 50 mV and **e** isoflurane inhibition (2 mM) of structured loop domain mutants. **f** Exemplary current traces of THIK1 structured loop domain mutants, prior to (black) and after (blue) application of 2 mM Isoflurane. For all panels, values and statistics are listed in Table S1 & S2 and error bars represent mean ± SEM

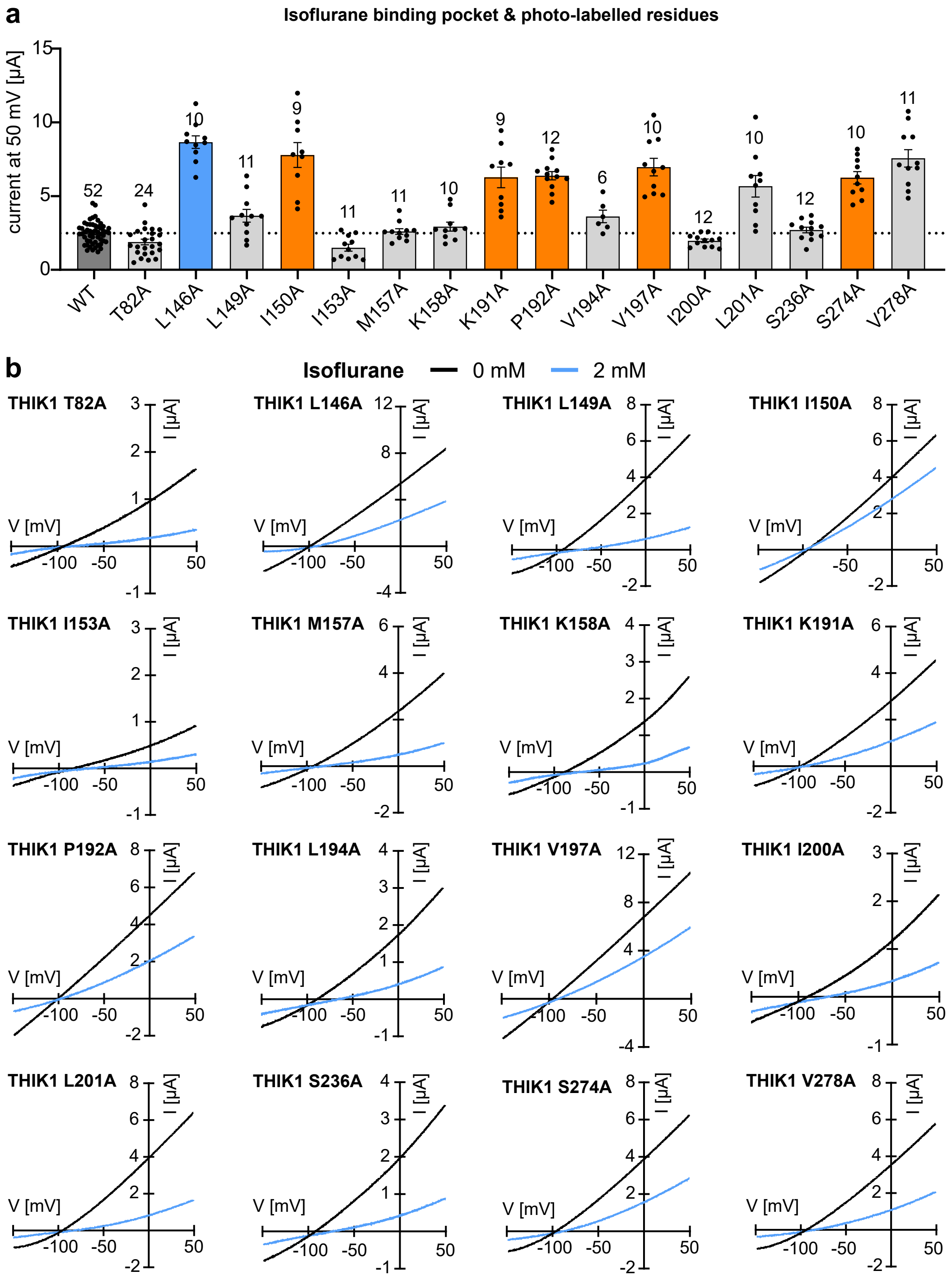

Fig S7. Alanine scanning of residues surrounding the isoflurane binding site. a Basal current value at 50 mV for THIK1 WT or alanine substitution mutant channels. The L146 azi-isoflurane photolabeled residue is highlighted in blue and residues found to impact isoflurane sensitivity (as shown in Fig. 4*E*) are in orange. Values and statistics are listed in Table S1 & S2 and error bars represent mean ± SEM **b** Exemplary current traces (TEVC, *Xenopus laevis*) of THIK1 T82A, L146A, L149A, I150A, I153A, M157A, K158A, L194A, V197A, I200A, L201A, S236A and V278A mutants before (0 mM, black) and after application of 2 mM Isoflurane (blue).

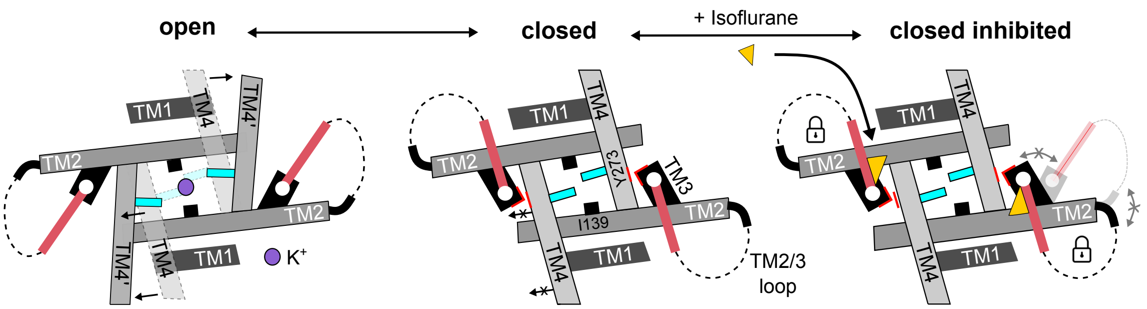

Fig S8. A speculative model of THIK1 gating and isoflurane inhibition. A cartoon of THIK1 gating. The closed state of the THIK1 channel (center) is stabilized by the structured region of the distal TM2/3 loop (red), which draws TM3 inward and restricts movement of TM4, narrowing the pore and locking the pore gate in a closed conformation. We anticipate that THIK1 opening (left) should involve movement of the structured TM2/TM3 loop to allow TM4 to rotate or translate, widening the pore at the Y273 gate to allow potassium ions (purple spheres) to flow through the channel. In the presence of isoflurane (yellow, triangle), the energetically favorable binding of the lipophilic anesthetic drug to the hydrophobic pocket formed by TM2, TM3, TM4, and the structured TM2/TM3 loop (right) stabilizes the closed gate conformation to reduce THIK1 opening.

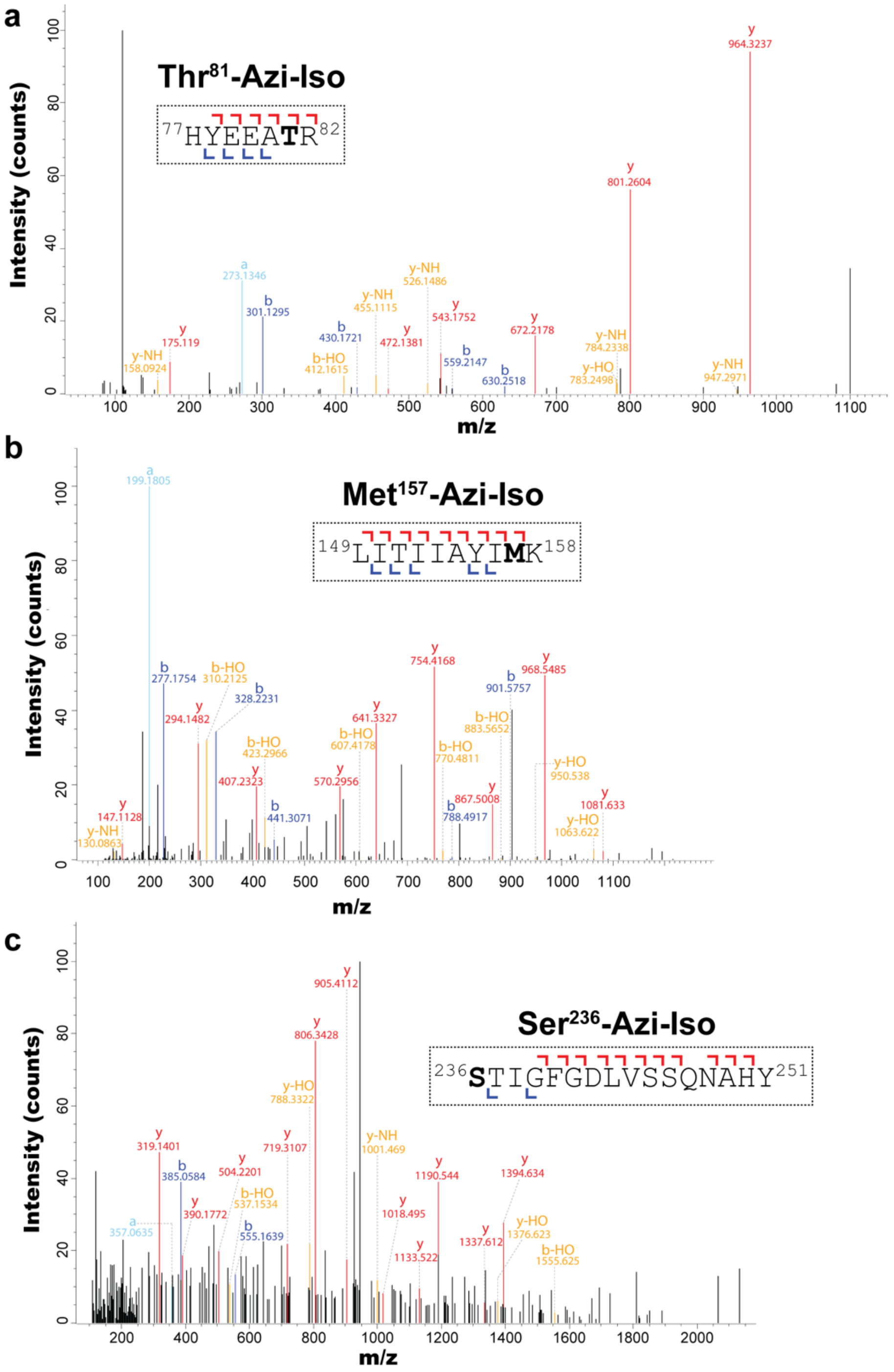

Fig S9. Mass spectrometry profiles for azi-isoflurane adducted peptides Profiles for **a** Thr81 adducted peptide, **b** Met157 adducted peptide, and **c** Ser236 adducted peptide. In all profiles, the relevant a, b, and y peptide peaks used for peptide identification are colored and labeled. The amino acid sequence of each identified peptide fragment is shown above the spectrum, with experimental fragmentation pattern displayed (*a,b* peptide fragments below in blue, *y* peptide fragments above in red).

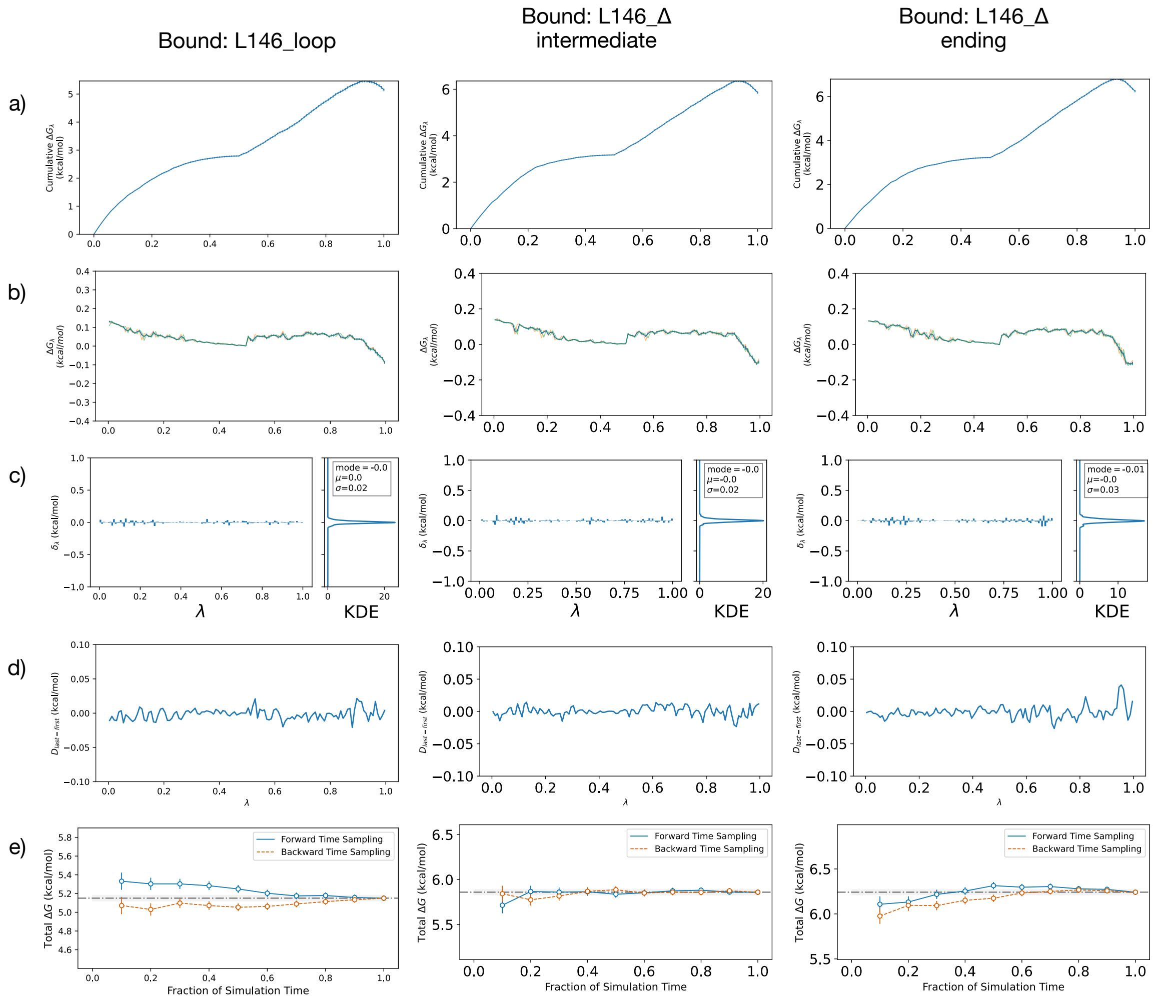

Fig S10. Convergence of free energy MD simulations of isoflurane in THIK1. The protein-bound calculations are shown in this figure, while bulk calculations are shown in Fig. S11. **a** Cumulative sum of Gibbs free energy ΔG for each window in the free energy perturbation calculation. **b** Per-window ΔG, not summed. Small per-window values suggest that within the window, the initial and final states were relatively similar. **c** Discrepancy in ΔG for each window, between forward (λ increasing) and backward (λ decreasing) directions. A kernel density estimation (KDE) of the probability distribution of these values is shown on the right portion of this subpanel. Smaller values imply better convergence. **d** Discrepancy in ΔG between first and last half of samples for each window. Smaller values imply better convergence. **e** Convergence plot, depicting what fraction of simulation time (x-axis) is necessary to achieve a particular magnitude of discrepancy between forward and backward sampling (y-axis). Note that the y-axis is not standardized between plots, such that detail in the curve is not lost.

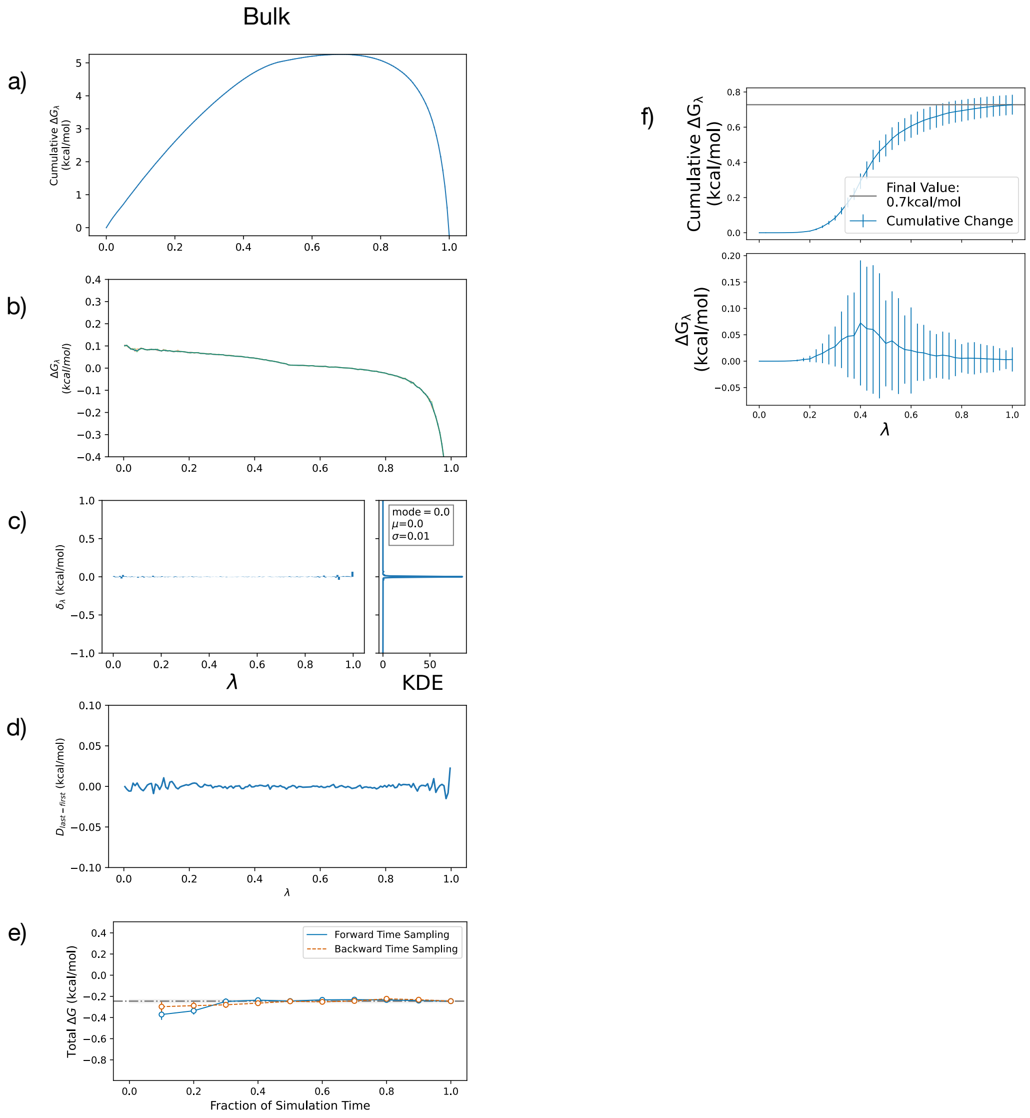

Fig S11. Convergence of free energy MD simulations of isoflurane in the bulk (aqueous) phase. See caption for Fig. S10 for description of panels **a-e**. Cumulative and individual ΔG for the thermodynamic integration (TI) calculation to calculate the energetic cost of the DBC restraint on the isoflurane is shown in **f**. A 5 Å DBC threshold was used.

Table S1. THIK1 WT and mutant channel basal current experiments.

Currents at 50 mV (TEVC, Xenopus laevis)						
**hTHIK1**	**n**	**Mean**	**SD**	**SEM**	**one-way ANOVA***	**P Value**
standard constructs						
WT	52	2.48	0.74	0.10	-	-
EM	20	6.64	1.66	0.37	****	<0.0001
cas8	13	7.56	1.61	0.45	****	<0.0001
intracellular gate						
Y273C	12	5.67	0.89	0.26	****	<0.0001
Y273F	32	5.60	1.38	0.24	****	<0.0001
Y273E	26	7.04	1.80	0.35	****	<0.0001
Y273A	22	9.69	1.29	0.28	****	<0.0001
Y273T	21	11.00	1.74	0.38	****	<0.0001
Y273S	10	10.40	1.36	0.43	****	<0.0001
I139A	10	9.59	1.99	0.63	****	<0.0001
N143A	21	3.01	0.89	0.19	ns	0.9833
N277A	21	5.49	1.06	0.23	****	<0.0001
M2/M3 loop						
TM2/3_dt	22	3.16	0.84	0.18	ns	0.666
TM2/3 GS+	12	2.68	0.60	0.17	ns	>0.9999
TM2/3 GS-	15	5.48	1.25	0.32	****	<0.0001
G189A	10	2.78	0.36	0.11	ns	>0.9999
W190A	14	2.57	0.68	0.18	ns	>0.9999
K191A	9	6.27	2.10	0.70	****	<0.0001
P192A	12	6.38	0.98	0.28	****	<0.0001
P192W	11	7.36	2.68	0.81	****	<0.0001
P192I	10	5.48	1.71	0.54	****	<0.0001
P192G	11	6.97	1.82	0.55	****	<0.0001
S193A	12	7.53	1.75	0.51	****	<0.0001
S193D	11	7.58	1.66	0.50	****	<0.0001

Currents at 50 mV (TEVC, Xenopus laevis)						
**hTHIK1**	**n**	**Mean**	**SD**	**SEM**	**one-way ANOVA***	**P Value**
Azi-Isoflurane photolabled residues						
T82A	24	1.88	0.94	0.19	ns	0.8411
L146A	10	8.66	1.36	0.43	****	<0.0001
M157A	11	2.60	0.62	0.19	ns	>0.9999
S236A	12	2.70	0.66	0.19	ns	>0.9999
Isoflurane binding site						
L149A	11	3.66	1.42	0.43	ns	0.1111
I150A	9	7.79	2.53	0.84	****	<0.0001
I153A	11	1.50	0.78	0.23	ns	0.5483
K158A	10	2.93	0.97	0.31	ns	>0.9999
V194A	6	3.61	1.04	0.43	ns	0.6916
V197A	10	6.97	1.88	0.59	****	<0.0001
I200A	12	1.97	0.41	0.12	ns	>0.9999
L201A	10	5.67	2.31	0.73	****	<0.0001
S274A	10	6.24	1.33	0.42	****	<0.0001
V278A	11	7.56	1.96	0.59	****	<0.0001
lipid pocket lining residues						
R92A	6	2.27	0.64	0.26	ns	>0.9999
G105I	17	6.11	1.37	0.33	****	<0.0001
R258A	10	2.41	0.69	0.22	ns	>0,9999
F262S	10	1.30	0.40	0.13	ns	0.2364
glycosylation of cap residues						
N59Q	22	3.3	0.9	0.2	ns	0.2637
N65Q	21	3.5	0.7	0.2	ns	0.0587
N59Q N65Q	17	3.1	1.2	0.3	ns	0.9467

*Dunnett's multiple comparisons test, WT vs. mutant

Summary of mean values, SD, SEM and p values obtained from TEVC recordings of THIK1 WT or mutant channels. Oocytes were clamped at -80 mV and currents were recorded after a step to 50 mV. Statistical significance was determined by one-way ANOVA followed by a Dunnett's multiple comparison test against THIK1 WT current values.

Table S2. THIK1 WT and mutant channel isoflurane inhibition experiments.

Isoflurane inhibition [%]						
**hTHIK1**	**n**	**Mean**	**SD**	**SEM**	**one-way ANOVA***	**P Value**
standard constructs						
WT	18	75.47	6.16	1.45	-	-
EM	10	64.77	7.65	2.42	*	0.0378
intracellular gate						
Y273C	12	5.97	3.64	1.05	****	<0.0001
Y273F	11	81.49	6.51	1.96	ns	0.80
Y273E	11	2.39	1.12	0.34	****	<0.0001
Y273A	10	11.27	3.11	0.98	****	<0.0001
Y273T	11	8.94	4.70	1.42	****	<0.0001
Y273S	10	6.59	1.10	0.35	****	<0.0001
I139A	10	6.46	3.50	1.11	****	<0.0001
N143A	10	35.74	10.21	3.23	****	<0.0001
N277A	11	56.93	13.59	4.10	****	<0.0001
TM2/TM3 loop						
TM2/3_dt	12	78.88	5.99	1.73	ns	>0.9999
TM2/3 GS+	12	74.68	5.25	1.52	ns	>0.9999
TM2/3 GS-	10	47.05	9.45	2.99	****	<0.0001
G189A	10	72.38	2.09	0.66	ns	>0.9999
W190A	12	61.41	9.57	2.76	****	<0.0001
K191A	10	62.70	9.05	2.86	**	0.0015
P192A	11	48.83	10.29	3.10	****	<0.0001
P192W	11	45.31	10.43	3.144	****	<0.0001
P192I	10	63.45	11.02	3.486	**	0.0086
P192G	11	42.55	12.53	3.779	****	<0,0001
S193A	12	52.49	10.57	3.05	****	<0,0001
S193D	11	64.75	12.16	3.67	*	0.0261

Isoflurane inhibition [%}						
**hTHIK1**	**n**	**Mean**	**SD**	**SEM**	**one-way ANOVA***	**P Value**
Azi-Isoflurane photolabled residues						
T82A	12	78.55	7.88	2.28	ns	>0.9999
L146A	10	53.59	12.10	3.83	****	<0.0001
M157A	15	79.28	4.86	1.25	ns	0.9991
S236A	11	78.08	2.77	0.83	ns	>0.9999
Isoflurane binding site						
L149A	11	79.91	6.35	1.92	ns	0.9967
I150A	12	30.55	8.84	2.55	****	<0.0001
I153A	11	72.62	9.04	2.73	ns	>0.9999
K158A	11	73.81	8.78	2.65	ns	>0.9999
V194A	5	76.32	5.39	2.41	ns	>0.9999
V197A	10	57.08	9.08	2.87	****	<0.0001
I200A	9	72.81	4.91	1.64	ns	>0.9999
L201A	10	73.92	4.26	1.35	ns	>0.9999
S274A	10	60.04	8.82	2.79	****	<0.0001
V278A	11	67.43	9.26	2.79	ns	0.2422
lipid pocket lining residues						
R92A	5	71.87	6.89	3.08	ns	>0.9999
G105I	9	74.95	7.62	2.54	ns	>0.9999
R258A	11	76.83	5.78	1.74	ns	>0.9999
F262S	10	77.92	5.91	1.87	ns	>0,9999

*Dunnett's multiple comparisons test, WT vs. mutant

Summary of mean values, SD, SEM and p values obtained from TEVC recordings of THIK1 WT or mutant channels. Oocytes were clamped at -80 mV and currents were recorded after a step to 0 mV, before and after application of 2 mM Isoflurane. Statistical significance was determined by one-way ANOVA followed by a Dunnett’s multiple comparison test against THIK1 WT current values.

Table S3. MD simulation data table.

MD Percent Occupancy (less than 8Å)		
THIK1 Residue #	L146_loop	L146_Δ
LEU146	36.80%	-
GLU147	74.10%	-
ILE150	100.00%	100.00%
THR151	87.20%	98.13%
ILE152	-	44.91%
ILE153	75.90%	99.81%
ALA154	93.10%	100.00%
TYR155	25.80%	64.71%
MET157	-	85.43%
LYS158	-	46.59%
LYS191	99.00%	89.26%
PRO192	100.00%	100.00%
SER193	99.80%	100.00%
VAL194	100.00%	100.00%
TYR195	87.80%	87.77%
TYR196	67.90%	94.21%
VAL197	100.00%	100.00%
MET198	96.20%	51.54%
SER274	56.90%	-
ASN277	85.90%	-
VAL278	100.00%	63.49%
ILE279	26.70%	-
ILE281	97.90%	33.71%

Isoflurane Binding Affinity Prediction			
FEP Result	L146_loop	L146_Δ intermediate	L146_Δ end
ΔG of Binding	-4.3 kcal/mol (SE: 0.1 kcal/mol)	-5.0 kcal/mol (SE: 0.1 kcal/mol)	-5.3 kcal/mol (SE: 0.1 kcal/mol)
Predicted K_D	794 µM (95% CI: 574–1100 µM)	249 µM (95% CI: 180–344 µM)	159 µM (95% CI: 109–209 µM)

Summary of equilibrium MD simulations of THIK1 in the presence of an isoflurane molecule placed near the L146 binding site. Percent occupancy of THIK1 residues by isoflurane (left) during two independent simulations, with the unstructured TM2/TM3 loop computationally modelled (L146_loop) or deleted (L146_Δ). Alchemical free energy perturbation results for calculations based on either the final isoflurane pose in the 146_loop simulation, an intermediate pose from the L146_Δ simulation, or the final pose from the L146_Δ simulation, showing calculated ΔG of binding and associated predicted K_D.
